# Rising mean incomes for whom?

**DOI:** 10.1371/journal.pone.0242803

**Published:** 2020-12-16

**Authors:** Liang Frank Shao, Melanie Krause

**Affiliations:** 1 School of Economics, Henan University, Kaifeng, Henan, China; 2 Department of Economics, Hamburg University, Hamburg, Germany; TED University, TURKEY

## Abstract

Not everybody is benefiting equally from rising mean incomes. We discuss the mean-income population share (*MPS*), the population percentage of earners below mean income, whose evolution can capture how representative rising mean values are for middle income households. Tracking *MPS* and its associated income share *MIS* over time indicates to what extent economic growth is inclusive of both the middle and the bottom of the income distribution. We characterize *MPS* and *MIS* analytically under different growth scenarios and compare their parametric estimation using micro-level and grouped income data. Our empirical application with panel data of 16 high- and middle-income countries shows that in the last decades rising mean incomes have mostly not favored middle income households in relative perspective, while the overall welfare effects of the changes in *MPS* and the correlation structure with the Gini coefficient are mixed.

## 1 Introduction

In recent decades, mean incomes have risen in most countries around the world, as national statistics show. But it is well-known that the benefits of economic growth are not distributed equally among the individuals involved in an economy: Across developing countries, there is a sizable heterogeneity to what extent economic growth reaches the poor and leads to falling poverty [see for instance 1, 2]. In many industrialized economies, the debate has in recent decades focused on the fate of the middle class [3, 4]. The concept of ‘inclusive growth’, which requires that the incomes of all individuals in the distribution jointly increase and share a fair proportion of the growth, has been made an official goal by the OECD [5, 6]. In a similar vein, the World Bank has prioritized the notion of ‘shared prosperity’ [7]. It has become clear that mean income is an imperfect welfare indicator to trace over time, if a growing mean income does not entail improvements in living standards for large parts of the population. While this is an active research area with many contributions [see for instance 8–10], there is an ongoing discussion about how to measure the inclusiveness of growth in a simple and transparent way.

This paper presents two intuitive measures linking the mean income to different parts of the distribution, and shows that their changes over time capture the inclusiveness of growth in terms of middle- and low-income individuals. In particular, the mean-income population share, *MPS* is defined as the percentage of individuals with an income less than the mean. At a given point in time, *MPS* yields insights about the distribution of income. We argue that *MPS* is especially useful in a dynamic setting: As it can only change when households move above or below the mean income point, it is particularly representative of middle income households. For example, if mean income rises over time, but the increase is more subdued for middle income households, more of them will have an income below the mean, which is reflected in a rise in *MPS*.

*MPS* is complemented by the mean income share, *MIS*, the corresponding portion of total income held by households below the mean. With *MPS* kept constant, increases in *MIS* can be interpreted as relative welfare improvements of households below mean income. The interplay of *MPS* and *MIS* allows us to analyze with two simple numbers how many people are benefiting from economic growth and to what extent: Changes in *MPS* capture whether more or less people are ranked below mean income, while changes in *MIS* express the magnitude of the relative income effects for these individuals.

We characterize *MPS* and *MIS* formally in terms of the income distribution function and the Lorenz curve. In various growth scenarios, we contrast the behavior of these two indices with inequality measures such as the Gini coefficient and distributional statistics such as the skewness. We demonstrate analytically that *MPS* exhibits unambiguous reactions to income growth at various parts of the distribution, which are neither reflected by the Gini coefficient nor the skewness. On the other hand, different combinations of *MPS* and *MIS* can lead to the same Gini coefficient, Pietra ratio (Hoover, Schutz, or Robin Hood index), or Bonferroni index. Looking at *MPS* and *MIS* can hence provide insights into which intradistributional movements lead to the observed changes in inequality.

We supplement our formal characterization with an empirical analysis of cross-country micro-level income data sets from LIS [11]. Looking at 16 high- and middle-income countries from the 1980s to the 2010s, we find that rising mean incomes have mostly not been representative of the middle and bottom incomes of the distribution. In fact, economic growth has led to increases in *MPS*, indicating that more households have moved from above to below the mean-income threshold of the income distribution. At the same time, *MIS* did not increase to the same extent, indicating that households below mean income have lost in relative terms. However, the co-movements of *MPS* with *MIS* vary across countries and years, as does their correlation with the Gini coefficient. Our results suggest that in countries with different labor market conditions and redistributive policies, economic growth has resulted in different welfare effects on relatively low income households. Yet, the squeeze of middle income households despite an overall economic expansion is a robust feature reflected in our analysis of *MPS*. This does not become clear using any summary measurements of inequality.

Our paper anchors *MPS* and *MIS* firmly in the income and growth literature, where they have been overlooked until now. With the exception of Shao [12], who provides an indirect inference procedure for disposable income *MPS* and *MIS* in the presence of sparse data, we have not found other applications of this concept in the literature. We argue that they are useful statistics for three reasons: (i) Their simple calculation and interpretation make them particularly meaningful for informing policymakers as well as the public debate about the distributional changes of economic growth on lower- and middle income households. Figures on growth in GDP per capita can be supplemented with changes in *MPS* and *MIS* to see who is relatively benefiting from these rising mean incomes. (ii) Their analytical tractability makes them good target statistics for researchers working with data of income distributions and Lorenz curves, because not all parametric Lorenz functions are equally flexible in capturing the empirical evolution of *MPS* and *MIS*. (iii) *MPS* and *MIS* provide a complementary perspective to inequality measures by respectively focusing on two dimensions of the distribution and showing growth effects on middle- and lower-income households that summary measures of income inequality cannot reflect.

More generally, our paper adds to the distributional literature that discusses the shortcoming of mean income as a measure of average living standard. For example, there is a tendency to look at the median in addition to the mean, whose recent empirical divergence has been duly documented [13]. However, mean income is arguably still the most widely used statistic. From the conceptual side, mean income can be well defined by continuous income density functions which facilitates theoretical operations in all cases, some of which are beyond the median’s capability. Furthermore, mean income rather than the median is the concept corresponding to GDP per capita in macroeconomic growth models [14, 15]. In order to see how GDP per capita growing along the balanced growth path translates into relative welfare changes for the whole distribution, we consider *MPS* and *MIS*.

Our work also relates to the literature strand of the growth effects on poverty. First, there is a huge literature on the measurement of pro-poor growth specifically [see for instance 8, 9, 16, 17], focusing exclusively on the lower end of the income distribution. For example, Kakwani and Son [18] define a poverty-equivalent growth rate by relating mean-income growth to its distributional effects between the poor and non-poor. What unites these measures is their concentration on growth effects on the poor, with no relation to changes for middle incomes around the mean of the distribution. Compared to that, the combination of *MPS* and *MIS* is a broader concept.

On the other hand, a lot has been written about the middle class, in particular in industrialized economies [3, 4]. There is neither a theoretical nor widely-accepted empirical definition of the middle class in the related literature [19, 20], and it is not the goal of this paper to provide one, even though changes in *MPS* may be related to the middle class. In particular, we argue that changes in *MPS* can be a simple but informative statistic to look at, when evaluating how households in the middle of the distribution are doing over time. The property that *MPS* only changes when households’ incomes move above or below the distributional mean makes it very sensitive to this particular portion of the distribution.

Moreover, growth incidence curves, as introduced by Ravallion and Chen [10] can provide a granular graphical representation of the extent to which each percentile of the population is benefiting from economic growth [21]. While these curves provide more detailed insights, the growth effects on *MPS* and *MIS* have the appeal of summarizing distributional information including the effects on both lower- and middle income households, and which are not directly reflected by growth incidence curves. Yet, our analysis lends itself to a generalization to other thresholds than the mean income point, which would then be directly related to growth-incidence curves. We explain this in more detail in our empirical study.

The remainder of this paper is structured as follows: In Section 2 we provide the analytical characterization of *MPS* and *MIS* as measures of growth inclusiveness and present the data for our empirical cross-country investigation. Section 3 discusses the results of the empirical investigation, and Section 4 concludes. Formal proofs as well as additional empirical statistics are contained in the S2 File.

## 2 Materials and methods

### 2.1 Definition of *MPS* and *MIS*

As the percentage of the population which has an income below the mean, *MPS* can be defined in terms of the cumulative income distribution function (CDF), *F*(*y*).

**Definition 1**. *Let* *y*_*i*_ *denote the ordered income for individual* *i*, *i* = 1, ‥*N*, *and* *μ* *the mean income*. *The mean population share*, *MPS*, *is the percentage value that the income CDF* *F*(*y*) *reaches at the mean μ*:

$$\begin{array}{c} MPS=F\left(\mu \right)=\frac{1}{N}\sum_{i=1}^{N}I_{y_{i}\leq \mu } \end{array}$$ (1)

*with* I *as the indicator function*.

*MPS* takes on values in (0, 1]. Note that *MPS* = 0 can only be reached asymptotically: If *N* − 1 individuals each have an income of *y*, and only one individual has a positive income of *a* < *y*, then *μ* > *a* and *MPS* = 1/*N*. As *N* → ∞, *MPS* → 0. *MPS* = 1 is attained in the extreme case of income equality. With income *y* = *μ* for all individuals, *F*(*μ*) = *MPS* = 1.

For a symmetric distribution, in which the mean and median coincide, it holds that *MPS* = 0.5. A positively (negatively) skewed distribution has an *MPS* above (below) 0.5, so that [0.5, 1] is the prevalent range for most empirical applications. Higher values of *MPS* indicate that more individuals have an income below *μ*. For instance, if the mean is lifted up by rich earners in the tail, there will be comparatively many individuals below the mean.

But *MPS* is not an inequality measure. It captures the share of individuals earning less than the mean, which is not representative of inequality of the whole distribution. For example, it is evident that *MPS* does not satisfy the Pigou-Dalton transfer principle [22]: An income transfer from a richer to a poorer individual on either side of the mean will leave *MPS* unaffected. Therefore, a high *MPS* value does not necessarily go in line with a high Gini coefficient. This becomes evident when considering the extreme case where 99% of the population have an income of *y* and 1% of individuals have an income of 2*y*. The mean *μ* = 1.01*y* is higher than *y* and therefore *MPS* = 0.99. So, 99% of the population have an income below the mean but the Gini coefficient is very low at 0.0098.

Closely related to *MPS* is the mean income share, *MIS*, which is the share of total income that the individuals below the mean hold. It can be computed as

$$\begin{array}{c} MIS=\frac{\sum_{i=1}^{N}y_{i}\cdot I_{y_{i}\leq \mu }}{\sum_{i=1}^{N}y_{i}}=\frac{\sum_{i=1}^{\left[MPS\cdot N\right]}y_{i}}{\sum_{i=1}^{N}y_{i}}=MPS\cdot \frac{\mu_{sub}}{\mu }, \end{array}$$ (2)

where [*x*] denotes the integer part of the number *x*, and $\mu_{sub}=\frac{1}{\left[MPS\cdot N\right]}\sum_{i=1}^{N}y_{i}\cdot I_{y_{i}\leq \mu }$ is the mean of all incomes below the mean *μ*. *MIS* can also take on values in (0, 1], but it is smaller than 0.5 in positively skewed income distributions which are empirically relevant. *MPS* simply counts individuals below the mean, whereas *MIS* supplements this figure with the respective incomes of the individuals. A higher *MIS* ceteris paribus suggests that individuals below the mean are holding a larger share of total income. Together, *MPS* and *MIS* provide a full perspective of the relation between mean income and various parts of the distribution. It is important to note the focus of the two measures on different parts of the distribution when tracking changes over time: In a dynamic setting, *MPS* is particularly sensitive to changes in the middle of the income distribution; only individuals moving above or below the mean have a direct impact on *MPS* according to Definition 1. By contrast, *MIS* is equally sensitive to all individuals at the bottom and middle of the distribution, as long as their incomes *y*_*i*_ are below the mean, see (2).

This characterization suggests that *MPS* and *MIS* are informative statistics for cross-country comparisons on relative welfare. Looking only at mean income neglects that in some countries a vast majority of individuals might be poorer, so that this number does not capture their living standards. *MPS* and *MIS* help to put mean income as a welfare indicator into a relative perspective to the income distribution. Also, while inequality measures such as the Gini coefficient do not distinguish between top- and bottom- inequality, *MPS* and *MIS* focus specifically on individuals below the mean compared to those above. This is why two different distributions might have the same Gini coefficient but very different *MPS* and *MIS*.

**Fig 1:**
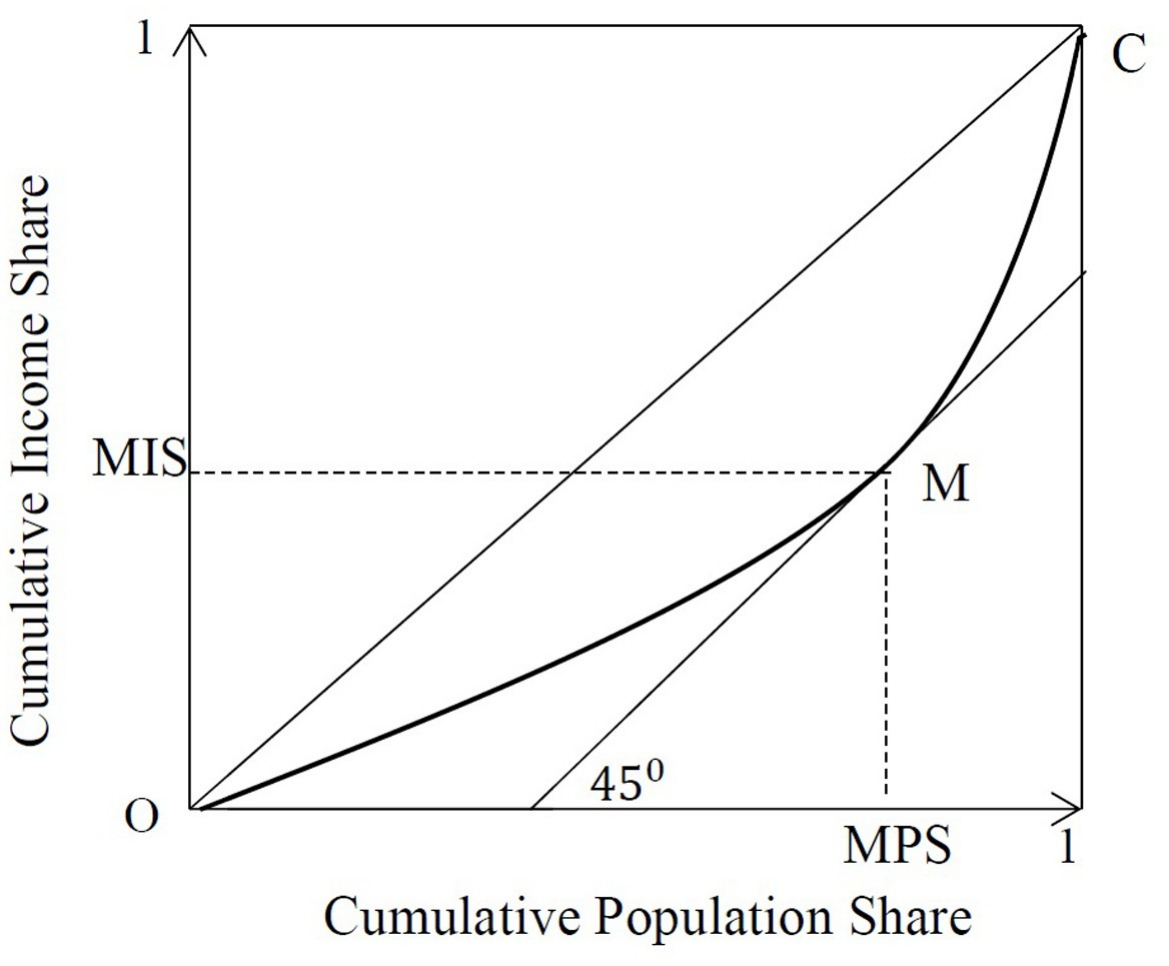
Illustration of *MPS* and *MIS* on the LC.

Still, there are connections between the two measures and inequality indices: For a given *MPS* value, a higher *MIS* goes along with a lower Gini coefficient, because of lower bottom inequality. For a given *MIS*, a higher *MPS* will be associated with a higher Gini coefficient because it implies that the mean is driven up by rich earners. Furthermore, the difference between *MPS* and *MIS* can also be used as an inequality measure; it is equivalent to the Pietra Index (also called the Hoover index, Robin Hood index, or the Schutz index). The Pietra Index can be interpreted as the income share that would have to be redistributed to achieve an egalitarian distribution. It is defined as the maximum vertical deviation between the Lorenz curve and the egalitarian line, $P=\max_{0\leq p\leq 1}\left[p-L\left(p\right)\right]$. Sarabia [23] shows that this holds at *y* = *F*(*μ*). By Definition 1, this is *MPS*. With *MIS* = *L*(*MPS*), it follows that *P* = *MPS* − *MIS*. These relationships are illustrated in Fig 1. For the Pietra index as a measure of intradistributional income inequality, see [24]. Also, it is possible to rewrite the Bonferroni index as $1-\frac{MIS}{MPS}$ in the special case of a triangular Lorenz curve. This shows that it is the interplay of *MPS* and *MIS* that drives various measures of income inequality. Different combinations of *MPS* and *MIS* might yield the same inequality in terms of the Gini coefficient, Pietra or Bonferroni index. However, tracing *MPS* and *MIS* allows us to look below the surface to see what is driving changes in inequality, as we will see in the following.

### 2.2 Inclusiveness of income growth

An analysis of *μ*, *MPS*, and *MIS* at any point in time is insightful for measuring relative living standards and for analyzing how institutional differences across countries are influencing different parts of the income distribution. However, even more relevant for policymakers is tracing *MPS* and *MIS* over time, in particular in periods of economic growth. In the following theorem, we derive that *MPS* shows unambiguous changes in reaction to growth at various parts of the income distribution.

**Theorem 1**. *Consider a population at time t* = 1 *with ordered incomes* *y*_*i*,1_, *i* = 1, …, *N*, *and mean income* *μ*_1_. *Define three growth scenarios from period 1 to 2 as follows*:

$$\begin{array}{ccc} pure\;top\;income\;growth: & & y_{i,2}=\{\begin{array}{c} y_{i,1},\;for\;i<\left[\left(1-p\right)N\right]\\ c\cdot y_{i,1},\;for\;i\geq \left[\left(1-p\right)N\right]\;with\;c>1,\;y_{[(1-p)N],1}>\mu_{1} \end{array}\\ pure\;bottom\;income\;growth: & & y_{i,2}=\{\begin{array}{c} c\cdot y_{i,1},\;for\;i\leq \left[pN\right]\;with\;c>1,\;y_{[pN],2}<\mu_{1}\\ y_{i,1},\;for\;i>\left[pN\right] \end{array}\\ pure\;middle\;income\;growth: & & y_{i,2}=\{\begin{array}{c} y_{i,1},\;for\;i<\left[lb\cdot N\right]\;or\;i>\left[ub\cdot N\right],\;y_{[ub\cdot N+1],1}>\mu_{2}\\ c\cdot y_{i,1},\;for\;\left[lb\cdot N\right]\leq i\leq \left[ub\cdot N\right]\;with\;c>1, \end{array} \end{array}$$

*where* 0 < *p* << 0.5 *is the population share to be affected by top or bottom income growth*, *lb* *and ub are the lower and upper bounds for incomes to be affected by middle income growth*. *MPS is then characterized by the following reactions in different growth scenarios*:

(a) Invariant to relative income changes by a factor c
(b) Invariant to absolute income changes by an absolute amount a
(c) *MPS (weakly) increases for the pure top income growth*: *MPS*_2_ ≥ *MPS*_1_
(d) *MPS (weakly) increases for the pure bottom income growth*: *MPS*_2_ ≥ *MPS*_1_
(e) *MPS (weakly) decreases for the pure middle income growth*: *MPS*_2_ ≤ *MPS*_1_

*Proof*. See S1 File.

Hence, if we have inclusive growth across the whole income distribution, both in relative and absolute terms (scenarios a and b), *MPS* stays constant. If increases in mean income are accompanied by changes in *MPS*, it indicates that some parts of the distribution are gaining more than others. The direction of the change in *MPS* expresses the relative welfare effects on middle income earners: If incomes at the top or bottom grow faster than at the middle of the income distribution, *MPS* increases (scenarios c and d); middle income households cannot keep up with the rising mean and some fall below it. Middle income growth, however, decreases *MPS* (scenario e), as more households will have an income that exceeds the mean. The middle income focus of *MPS* becomes also clear when decomposing its reaction into the sum of two effects: Income growth by the factor *c* of the affected individuals has a direct effect on *MPS*; the overall increase in *μ* has an indirect effect. In scenarios (c) and (d), *MPS* changes only due to the indirect effect. However, we have both a direct effect and an indirect effect—and typically a stronger reaction—if the middle of the distribution exhibits income growth as in (e).

Table 1 compares the reactions of *MPS* to those of *MIS*, the Gini coefficient, and the skewness for the above growth scenarios. Neither the Gini coefficient nor the skewness can capture the evolution of *MPS*. The Gini coefficient reacts differently to growth at the top and bottom, and is ambiguous towards middle income growth. The reactions of skewness often depend on the underlying distributions and the precise changes, while the changes in *MPS* hold in general. For example, if the initial distribution is lognormal and the top 10% (20%) of incomes double, the skewness increases (decreases). By contrast, *MPS* will always (weakly) rise because mean income has increased and some individuals above the old mean might fall below the new mean.

**Table 1:** Comparison of measures in the growth scenarios from theorem 1.

Description	*Measures after income growth*				
	Mean *μ*_2_	*MPS*_2_	*MIS*_2_	*Gini*_2_	Skewness *sk*_2_
(a) Uniform relative growth	*c* ⋅ *μ*_1_	*MPS*_1_	*MIS*_1_	*Gini*_1_	*sk*_1_
(b) Uniform absolute growth	*μ*_1_ + *a*	*MPS*_1_	> *MIS*_1_	< *Gini*_1_	*sk*_1_
(c) Top income growth	> *μ*_1_	≥ *MPS*_1_	⋚ *MIS*_1_	> *Gini*_1_	⋚ *sk*_1_
(d) Bottom income growth	> *μ*_1_	≥ *MPS*_1_	> *MIS*_1_	≤ *Gini*_1_	⋚ *sk*_1_
(e) Middle income growth	> *μ*_1_	≤ *MPS*_1_	⋚ *MIS*_1_	⋚ *Gini*_1_	⋚ *sk*_1_

*Notes*: The table shows how mean income, *MPS*, *MIS*, Gini coefficient $\frac{E|y_{i}-y_{j}|}{2\mu }$, and skewness $E[{\left(\frac{y-\mu }{\sigma }\right)}^{3}]$ react in the income growth scenarios defined in Theorem 1 in S1 File. For *MIS*, the derivations can be found in S1 File.

When we observe heterogeneous changes across the income distribution rather than growth affecting just one part, the co-movements between *MPS* and *MIS* become important. On its own, *MIS* does not exhibit unambiguous reactions in all growth scenarios (Table 1), unlike *MPS*. But together, the two measures can express which part of the distribution is benefiting in particular. While changes in *MPS* are driven by middle income earners, its combination with *MIS* can reflect what is happening to all individuals below the mean. The relative welfare changes of these individuals following mean income growth, as captured by the joint reactions of *MPS* and *MIS*, are shown in Table 2, a 3 × 3 welfare matrix. All effects should be compared to the baseline scenario of inclusive income growth (cell (2,2)), which is distribution-neutral and keeps *MPS* and *MIS* constant. If a constant *MPS* goes in line with an increasing (decreasing) *MIS*, individuals below the mean gain more (less) from economic growth than individuals above. Also, if more middle income individuals move above the mean (so that *MPS* decreases), but *MIS* stays constant (cell (3, 2)), those below the mean are better off because the same income share is now accumulated by fewer individuals. Obviously, the most strongly pro-poor and pro-middle income growth would be reflected in a decreasing *MPS* and simultaneously increasing *MIS* (cell (3,1)). Note that the welfare effects are in line with the effects on the Pietra ratio (*MPS* − *MIS*).

**Table 2:** Relative welfare effects of *MPS*&*MIS* comovements.

Welfare effects	MIS		
	↑	→	↓
↑	↕	↓	↓↓
MPS →	↑	→	↓
↓	↑↑	↑	↕

*Notes*: The table shows how the reactions of *MPS* and *MIS* to a rising mean income can be interpreted in terms of relative welfare effects for individuals below mean income (as compared to those above the mean). ↑↑ and ↑ (↓↓ and ↓) denote very strong and strong increases (decreases) in relative welfare, respectively. → expresses that welfare increases in line with the rest of the distribution (inclusive growth). ↕ denotes ambiguous welfare effects.

### 2.3 *MPS* and *MIS* of parametric lorenz functions

With micro-level income data, one can simply compute *MPS* and *MIS* with the definitions above. In many cross-country applications, however, only grouped-level percentile data are available. Then one can exploit the analytical tractability of *MPS* and *MIS* in terms of the Lorenz curve (LC), which links the cumulative income share *L* to the cumulative population share *p*.

**Theorem 2**. *For a continuously differentiable LC*, *L*(*p*), *MPS as defined in Definition 1 is the value p at which the first derivative of the LC equals unity*:

$$\begin{array}{c} L^{\prime }\left(MPS\right)=1 \end{array}$$ (3)

*Proof*. The LC and the income CDF are related by the following formula [see 25, 26]:

$$\begin{array}{c} F^{-1}\left(p\right)=L^{\prime }\left(p\right)\cdot \mu . \end{array}$$ (4)

At the value *p* = *MPS*, we can use Definition 1 and take the inverse of the function

$$\begin{array}{c} F^{-1}\left(MPS\right)=\mu . \end{array}$$ (5)

By combination of (4) and (5), it follows that (3) holds.

Hence, *MPS* is located at the point where a 45 degree line is tangential to the LC, see Fig 1. Uisng the definition of *MIS* as the income share of all individuals below the mean, *MIS* can be written as the Lorenz curve ordinate of *MPS*:

$$\begin{array}{c} L(MPS)=MIS \end{array}$$ (6)

In empirical studies with grouped data, parametric Lorenz functions, such as the lognormal or the Pareto LC, are fitted to the percentile points. Theorem 2 allows to derive the *MPS* and *MIS* values that parametric LCs imply. In Table 3 we list the closed forms for *MPS* and *MIS* for the most-widely used uni-parametric and multi-parametric functional forms. For example, for a lognormal distribution with parameter *σ* > 0, we have $MPS=\Phi (\frac{1}{2}\sigma )$, where Φ(.) denotes the cumulative normal distribution. With varying *σ* values, *MPS* can take on values on the whole realm (0.5, 1). But not all parametric forms are equally able to capture changes in *MPS* and *MIS* and therefore express the inclusiveness of economic growth. The Weibull LC, although known for its flexibility in fitting LCs associated with both unimodal and zero-modal income densities [27], can only represent *MPS* ∈ [0.43, 0.64]. In the following section we are going to examine the importance of these limitations and explore which parametric forms can best trace the recent empirical evolution of *MPS* and *MIS*.

**Table 3:** *MPS* and *MIS* for widely-used Lorenz curves.

LC name and parameters	*L*(*p*)	*MPS*	*MPS* Range	*MIS* = *L*(*MPS*)	*MIS* Range
*Uni-parametric functions*					
Lognormal (*σ* > 0)	Φ(Φ^−1^(*p*) − *σ*)	$\Phi (\frac{1}{2}\sigma )$	(0.5,1)	$\Phi (-\frac{1}{2}\sigma )$	(0,0.5)
Pareto (*α* > 1)	$1-{\left(1-p\right)}^{1-\frac{1}{\alpha }}$	$1-(1-\frac{1}{\alpha }){)}^{\alpha }$	(0.625,1)	$1-(1-\frac{1}{\alpha }){)}^{\alpha -1}$	(0,0.625)
Weibull (*b* > 1)	$1-\frac{\Gamma (-log\left(1-\pi \right),1+\frac{1}{b})}{\Gamma (1+\frac{1}{b})}$	$1-e^{-\Gamma {\left(1+\frac{1}{b}\right)}^{b}}$	(0.43,0.64)	$1-\frac{\Gamma ({\left[\Gamma \left(1+\frac{1}{b}\right)\right]}^{b},1+\frac{1}{b})}{\Gamma (1+\frac{1}{b})}$	(0.26,0.43)
Chotikapanich [28] (*k* > 0)	$\frac{e^{kp}-1}{e^{k}-1}$	$\frac{1}{k}log[\frac{e^{k}-1}{k}]$	(0.5,1)	$\frac{1}{k}-\frac{1}{e^{k}-1}$	(0,0.5)
Rohde [29] (*β* > 1)	$p\frac{\beta -1}{\beta -p}$	$\beta -\sqrt{\beta (\beta -1)}$	(0.5,1)	$1-\beta +\sqrt{\beta (\beta -1)}$	(0,0.5)
*Multi-parametric functions*					
Kakwani [26] (*α* > 0, *δ*, *β* ∈ (0, 1)	*p* − *αp*^*δ*^(1 − *p*)^*β*^	$\frac{\delta }{\delta +\beta }$	(0,1)	$\frac{\delta }{\delta +\beta }-\alpha {\left(\frac{\delta }{\delta +\beta }\right)}^{\delta }{\left(\frac{\beta }{\delta +\beta }\right)}^{\beta }$	(0,1)
Wang and Smith [30]					
(*α* ∈]0, 1[, *α* + *β* > 0)	$\frac{1-\alpha }{1+\beta }\frac{1+\beta p}{1-\alpha p}p$	$\frac{1-\sqrt{1-\alpha }}{\alpha }$	(0.5,1)	$\frac{1+\beta (\frac{1}{\alpha }-\frac{1}{\alpha }\sqrt{1-\alpha })}{1+\beta }\cdot [1-\frac{1}{\alpha }+\frac{1}{\alpha }\sqrt{1-\alpha }]$	(0,0.5)
Villaseñor and Arnold [31]					
(*d* ≥ 0, *a* + *d* ≥ 1, *α* = *b*^2^ − 4*a* < 0,	0.5[−(*bp* + *e*)−				
*β* = 2*be* − 4*d*, *e* = −(*a* + *b* + *d* + 1) < 0	(*αp*^2^ + *βp* + *e*^2^)^0.5^]	see below	(0,1)	see below	(0,1)

*Notes*: $\Gamma \left(\alpha \right)=\int_{0}^{\infty }t^{\alpha -1}e^{-t}dt$ is the Gamma function. *MPS* for the Villaseñor and Arnold [31] Elliptical function is $\frac{-\beta \pm \sqrt{\beta^{2}-4\alpha \left(d-be-e\right)}}{2\alpha }$ with $b\geq -(a+d+\frac{a}{a+d})$, the corresponding *MIS* is $0.5(b\frac{\beta \pm \sqrt{\beta^{2}-4\alpha \left(d-be-e\right)}}{2\alpha }-e-\sqrt[{2}]{-d-e(a+d)})$

### 2.4 Data for the empirical investigation

In our empirical investigation, we apply the methods presented above in a cross-country setting. Looking at empirical *MPS* and *MIS* values over time, our goal is to investigate whether recent economic growth has been relatively inclusive of individuals ordered below mean income. We trace *MPS* and *MIS* over four decades (1978-2016) for different countries, analyze their comovements with changes in mean income, and study to what extent the parametric forms from Section 2.3 can capture these developments. Our data are harmonized cross-country micro-level income data sets from LIS [11]. To examine the evolution of the statistics, a long time span with frequent reporting periods is crucial. We therefore settle on those 16 high- and middle income countries which report income data for all waves between at least the 2nd (around 1985) and the 9th wave (around 2014). Note that the precise years vary across countries; the S2 File provides an overview. We work with both total and disposable household income. Both measures are calculated as equivalized income, which is obtained by dividing the household income by the square root of the number of household members. We follow the literature in weighting observations by the number of household members times household weights.

## 3 Results and discussions

### 3.1 Micro data: *MPS* and *MIS* across countries and time

The 16 countries in our data set are listed in Table 4 together with their *MPS* and *MIS* in the first and last available observation year. *MPS*, *MIS*, and the Gini coefficient for all countries and years are provided in the S2 File. The countries are known to differ in their levels of income inequality and social mobility [32], labor market structures [33] as well as societal attitudes towards redistribution and taxation [34]. Consequently, we observe a lot of cross-country variation in terms of *MPS* and *MIS*. For the last available year (around 2014), *MPS* ranges from 0.53 (the Netherlands) to 0.70 (Mexico).

**Table 4:** Summary statistics of the data set.

Country	Year		MPS		MIS		Mean growth p.a.	
	First	Last	First	Last	First	Last	Nom.	Real
AU	1981	2014	0.5952	0.6878	0.3564	0.3908	0.0527	0.0177
CA	1981	2013	0.6257	0.6283	0.3855	0.3619	0.0391	0.0131
DE	1978	2015	0.5815	0.6434	0.3887	0.3813	0.0243	0.0164
DK	1987	2013	0.6091	0.6641	0.3876	0.4368	0.0308	0.0114
ES	1980	2013	0.6315	0.6187	0.3987	0.3458	0.0695	0.0154
FI	1987	2013	0.5039	0.5578	0.3432	0.3585	0.0361	0.0156
IL	1979	2016	0.5605	0.6275	0.3232	0.3438	0.0553	0.0174
IT	1986	2014	0.5944	0.5778	0.3778	0.3538	0.0342	0.0075
LU	1985	2013	0.5348	0.5824	0.3825	0.3646	0.0524	0.0276
MX	1984	2012	0.6347	0.7026	0.3350	0.3464	0.2006	0.0077
NL	1983	2013	0.6219	0.5322	0.4094	0.3367	0.0321	0.0174
NO	1979	2013	0.4837	0.6542	0.3193	0.4301	0.0631	0.0189
PL	1986	2016	0.6151	0.6426	0.4096	0.4255	0.2328	0.0410
TW	1981	2016	0.6089	0.6591	0.4196	0.4345	0.0435	n.a.
UK	1979	2013	0.6002	0.6876	0.3789	0.4040	0.0563	0.0171
US	1979	2016	0.6128	0.6709	0.3542	0.3531	0.0408	0.0159

*Notes*: *MPS* and *MIS* are computed from LIS data. Nominal growth data are based on LIS household income survey means in national currency; the real growth data are based on real GDP per capita data from the World Bank’s World Development Indicators. The latter are unavailable for Taiwan. Both growth rates are computed from the sample beginning and end values at an annualized rate (except for Polish real data, which is based on the second rather than first year due to a missing observation).

While a detailed analysis of policy differences between these countries is beyond the scope of our analysis, we shed some light by conducting a crude grouping of countries: anglo-saxon countries, nordic countries, mediterranean countries as well as the diverse remaining group. Table 5 shows that, as expected, *MPS* is highest in anglo-saxon countries. With mean incomes driven up by top earners, there is a rather high share of individuals below the mean. By contrast, *MPS* is lowest in the nordic countries, with the mediterranean countries falling in between these two groups. The nordic countries also have the highest *MIS* out of the four groups: Despite their relatively low share of individuals below the mean, these households hold a larger share of total income than the more numerous below-mean income earners in other countries.

**Table 5:** *MPS* and *MIS* by country group.

	Anglo	Nordic	Med	Rest
*MPS*	0.6489	0.5876	0.6173	0.6263
(Std)	(0.0303)	(0.0574)	(0.0225)	(0.0426)
Obs	31	25	32	87
*MIS*	0.3662	0.3874	0.3652	0.3854
(Std)	(0.0206)	(0.0373)	(0.0253)	(0.0283)
Obs	31	25	32	87

*Notes*: The table summaries *MPS* and *MIS* over all available years for countries in a respective group: anglo-saxon (AU, CA, UK, US), nordic (DK, FI, NO), mediterranean (ES, IL, IT), and the diverse group of remaining countries (DE, LU, MX, NL, PL, TW).

**Fig 2:**
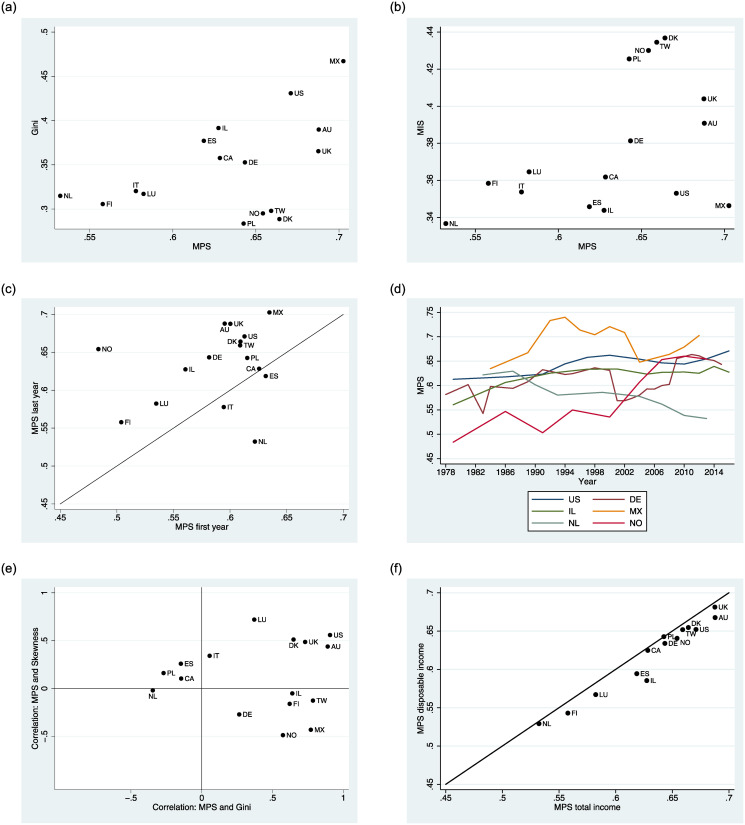
*MPS* and other statistics across countries and time.

It is intuitive to expect a positive cross-country correlation between *MPS* and income inequality. However, the scatter plot between *MPS* and the Gini coefficient in Fig 2a shows a more complex pattern. Despite a number of countries close to the diagonal line, we also find a group of countries with low income inequality, in particular, Norway, Poland, and Taiwan, which have comparatively high *MPS* values. They have few relatively poor households, while mean income is driven up by rich households, so that many middle income households are located below the mean. This is confirmed by their rather high *MIS* of 0.42-0.44. Hence, households below the mean hold a larger share of total income than in many others countries, both egalitarian and inegalitarian, see Fig 2b. For example, the U.S. and Denmark show similar levels of *MPS* of 0.60-0.61, but these households hold a total income share of 0.43 in Denmark and only 0.35 in the U.S. When countries differ in their *MIS*, a welfare ranking is only possible when taken together with *MPS*. The low *MIS* of Finland and the Netherlands is due to their low *MPS*, so that the corresponding income shares are accumulated by fewer individuals.

How has *MPS* evolved over the last three decades as mean incomes grew substantially? Fig 2c illustrates that *MPS* was higher in the last (ca. 2014) than in the first observation period (ca. 1981) for 13 out of 16 countries. The evolution of *MPS* over time in Fig 2d shows notable increases in many countries, with the only sustained decrease occurring in the Netherlands. These results suggest that mean incomes have become less representative of the middle of the distribution in most countries, as more middle income earners than before are now ordered below the national mean. Momentous structural changes have occurred during these decades, which have all impacted the income distribution of industrialized economies, most prominently along with skill-biased technological change [35, 36] and globalization [37, 38]. Top incomes have risen substantially in many countries [39, 40]. The literature has highlighted that the middle class, by contrast, has often been squeezed by wage polarization [41, 42]. Our results on the rise in *MPS* should be seen in this context: They reflect that many middle income households have been unable to keep up with rising mean income and have fallen behind in relative terms.

While changes in *MPS* are driven by the middle of the distribution, the co-movement with *MIS* can give an indication of the relative welfare effects for all households below the mean, including the poorest. Table 4 shows that *MIS* slightly increased for more than half of the countries from the beginning to the end of the sample period, but the changes are mostly small. Despite rising *MPS* and hence more individuals below the mean, their corresponding income shares have not tended to increase by much. The U.S. is a case in point: In 2016, 67% of individuals were below mean income rather than 61% in 1979. But their total income share stayed constant at 35%. Hence, in the U.S. not only the middle of the distribution, as shown by *MPS*, but more generally all individuals below the mean were gaining less from economic growth than the top earners. In Norway, by contrast, both *MPS* and *MIS* have increased substantially: Middle income earners have lost out but the welfare effects on lower income earners are ambiguous, in line with the welfare matrix in Table 2.

Income growth at the top always tends to increase both *MPS* and income inequality, but the distributional movements of other growth scenarios may not, so that inequality and *MPS* do not always move in the same direction. Fig 2e looks at the correlations between *MPS* and the Gini coefficient as well as between *MPS* and skewness for each country over time. For example, in the U.S. and Australia, the development of *MPS* correlates strongly with both the Gini coefficient and the skewness of the distribution. But for many others, including Germany, Poland, and Canada, the evolution of *MPS* shows either no significantly positive or even a moderately negative correlation with the Gini coefficient and skewness, see Fig 2e. Obviously, growth in top incomes can only be part of the story. The developments of the last decades have affected different parts of the income distribution in different ways across countries: Countries with labor market conditions and redistributive policies in favor of the poor might keep inequality constant, but *MPS* still rises as middle income earners lose out in relative terms.

We find that the same conclusions from total income carry over to disposable income in most countries: The disposable income *MPS* is only marginally lower than the one for total income (Fig 2f); note that this scatter plot omits Mexico and Italy, where the data does not distinguish between total and disposable income. Policies that redistribute from the top to the bottom decrease the Gini coefficient, but they do not tend to have a large impact on households near the income mean and hardly affect *MPS*. We also calculate the difference of the average distributional metrics (*MPS*, *MIS*, *Gini*) in terms of gross and disposable income for each country, finding mean squared errors (MSE) of *MPS*, *MIS* and Gini between gross income and disposable income across countries to be, respectively, 0.014, 0.017, and 0.037. Hence, the difference between disposable and gross income is more than twice as large for the Gini coefficient as for *MPS* and *MIS*. Disposable income *MIS* is smaller than its gross income counterpart in 12 countries, while the relationship is reversed in Taiwan. Disposable Gini is always smaller than gross Gini—in line with the literature [43]—, and disposable *MPS* is smaller than gross *MPS* in all countries except Poland.

We dig deeper in S18 Table in the S2 File, where we provide growth rates of mean income *g*_*μ*_, the growth rate of mean income for all individuals below the mean, *g*_*subμ*_, as well as distributional metrics between the first and last available year. Note that there is a direct relation between the changes in these variables: With *μ* as national mean income and *μ*_*sub*_ as the average income for individuals included in *MPS*, (2) can be rewritten as

$$\begin{array}{c} \frac{\mu_{sub}}{\mu }=\frac{MIS}{MPS} \end{array}$$ (7)

In terms of growth rates, this yields

$$\begin{array}{ccc} \frac{\Delta \mu_{sub}}{\mu_{sub}}-\frac{\Delta \mu }{\mu } & = & \frac{\Delta MIS}{MIS}-\frac{\Delta MPS}{MPS} \end{array}$$ (8)

$$\begin{array}{ccc} g_{sub\mu }-g_{\mu } & = & g_{MIS}-g_{MPS} \end{array}$$ (9)

where *g*_*subμ*_ denotes growth of mean income for individuals below national mean income, and *g*_*μ*_, *g*_*MPS*_, *g*_*MIS*_ are the growth rates of *μ*, *MPS*, and *MIS*, respectively.

If the mean income of individuals at the bottom and middle of the distribution grows at a rate that is not slower than the growth rate of national mean income, the rising mean income will be unambiguously beneficial to individuals below the national mean income. It is worth mentioning that the equations above apply to any point on the Lorenz curve. Repeating the analysis with different thresholds than the mean, one can conduct a study that would be directly complementary to growth incidence curves.

In S18 Table in the S2 File we can see that the group-specific mean income of individuals below the national mean increased more than national mean income for both gross and disposable incomes in three countries (Denmark, Mexico, and Poland), while both *MIS* and *MPS* increased in Denmark and Poland, and decreased in Mexico. So, individuals below mean income were relatively better off in the three countries because their *MIS* increased more than *MPS* did. But in the other 13 countries *MPS* always increased by more than *MIS*, making individuals at the middle and the bottom of the distribution worse off, as discussed above. An interesting situation happened in Germany and the USA, where gross *MIS* growth was negative, but the growth of disposable *MIS*, *MPS* and Gini had become positive. One possible interpretation of this would be that government policies were effective in propping up the poorest earners, while those at the middle benefited less from redistributive policies and fell below mean income, leading to an increase in *MPS*.

S19 Table in the S2 File provides standard deviations of the 5-percentile income shares of each country over time, highlighting the dynamics at the top, bottom and middle of the distribution. We find that the largest variation always happened to the top 5% income share, and the second largest happened to either the bottom or the second-highest 5% income share. Meanwhile, there is also substantial variation around the middle of the distribution.

While the analysis so far considers summary statistics, let us now investigate the effect of mean income growth on *MPS* and other measures in a panel regression. For each country *i* and available year *t*, we compute the changes in distributional measure *M* from the previous available year. We regress it on the difference in log mean income between the same years. We measure log mean income both in nominal and real terms, using the LIS income means in national currency for the former and real GDP per capita data from the World Bank’s World Development Indicators for the latter. Table 4 shows the heterogeneity of annualized growth rates over countries, ranging from 0.75% in Italy to 4.10% in Poland in real terms. There is also a sizable variation over time. Controlling for country-fixed effects and time specifics either via a time trend or year-fixed effects, we estimate

$$\begin{array}{ccc} \Delta M_{it} & = & Growth_{it}+\alpha_{i}+c\cdot t+\epsilon_{it} \end{array}$$ (10)

$$\begin{array}{ccc} \Delta M_{it} & = & Growth_{it}+\alpha_{i}+\gamma_{t}+\epsilon_{it} \end{array}$$ (11)

where the dependent variable *M* is, respectively, *MPS*, *MIS*, the Gini coefficient, skewness, or the Pietra ratio, *MPS* − *MIS*.

Note that we consider both the distributional effects and growth effects on Δ*M* by including Δ*MPS* on the right hand side of Eqs (10) and (11). However, this means that one should be cautious about a causal interpretation of the coefficients, as changes in *MPS* and *MIS* occur simultaneously.

The panel regression results in Table 6 below confirm that mean income growth was partially associated with increases in *MPS* (Panels A and B). In the specifications where the coefficient is statistically significant, it is always positive. *MIS* shows a tendency for positive reactions to growth (Panels C and D), but the magnitude is more subdued. When controlling for changes in *MPS*, the reactions of *MIS* to income growth lose significance in all specifications. This suggests that *MPS* moves more strongly with income growth than *MIS*.

**Table 6:** Panel regressions of changes in *MPS* and *MIS* on mean income growth.

*Panel A: Nominal growth*	*Dependent variable*: Δ*MPS*			
*growth_nom*	0.007**	0.000	0.002	-0.001
	(0.003)	(0.004)	(0.004)	(0.004)
Δ*MIS*		1.043***		1.035***
		(0.125)		(0.119)
Adjusted R^2^	0.032	0.726	0.113	0.754
Country FE	Yes	Yes	Yes	Yes
Year FE	No	No	Yes	Yes
Time Trend	Yes	Yes	Yes	Yes
Observations	159	159	159	159
Countries	16	16	16	16
*Panel B: Real growth*	*Dependent variable*: Δ*MPS*			
*growth_real*	0.108**	0.013	0.146**	0.034*
	(0.045)	(0.015)	(0.061)	(0.017)
Δ*MIS*		1.044***		1.032***
		(0.130)		(0.119)
Adjusted R^2^	0.076	0.728	0.167	0.765
Country FE	Yes	Yes	Yes	Yes
Year FE	No	No	Yes	Yes
Time Trend	Yes	Yes	Yes	Yes
Observations	148	148	148	148
Countries	15	15	15	15
*Panel C: Nominal growth*	*Dependent variable*: Δ*MIS*			
*growth_nom*	0.06***	0.002	0.003	0.002
	(0.002)	(0.003)	(0.004)	(0.003)
Δ*MPS*		0.689***		0.700***
		(0.078)		(0.073)
Adjusted R^2^	0.012	0.720	0.072	0.743
Country FE	Yes	Yes	Yes	Yes
Year FE	No	No	Yes	Yes
Time Trend	Yes	Yes	Yes	Yes
Observations	159	159	159	159
Countries	16	16	16	16
*Panel D: Real growth*	*Dependent variable*: Δ*MIS*			
*growth_real*	0.092**	0.018	0.109*	0.007
	(0.043)	(0.016)	(0.058)	(0.025)
Δ*MPS*		0.678***		0.698***
		(0.080)		(0.080)
Adjusted R^2^	0.057	0.723	0.115	0.750
Country FE	Yes	Yes	Yes	Yes
Year FE	No	No	Yes	Yes
Time Trend	Yes	Yes	Yes	Yes
Observations	148	148	148	148
Countries	15	15	15	15

*Notes*: Standard errors clustered at the country level are in parentheses.

Significant at:

* *p* < 0.10,

** *p* < 0.05,

*** *p* < 0.01.

The S2 File considers the results of three robustness checks and additional analyses. In particular, S20 Table in the S2 File reruns the panel regressions of changes in *MPS* and *MIS* on mean income growth, both contemporaneously and with a lag. With the lag we address the simultaneity issue. However, it is statistically insignificant in most specifications, yielding no evidence for a delayed impact of growth on *MPS* and *MIS*. The main coefficients tend to change very little with the inclusion of the lags.

In S21 Table in the S2 File, we rerun these regressions using the country group dummies of anglo-saxon, nordic and mediterranean countries defined in Table 5 as control variables instead of using country fixed effects. The results are remarkably similar, both qualitatively and quantitatively (for example, the coefficient for the effect of changes in *MIS* on *MPS* in Panel A has a coefficient of 1.049 rather than 1.043). This shows that country ideosyncracy as expressed by the fixed effects can to a large extent be captured by the country groups, which in turn, represent differences in the regulatory, labor market and welfare regimes.

Finally, in S23 Table in the S2 File we replace *MPS* or *MIS* as the dependent variable and instead consider the growth effects on different measures, namely the Gini coefficient, the skewness and Pietra ratio. The effects of growth on the Gini coefficient have different signs when nominal and real data are used but are never statistically significant. This heterogeneity of growth effects on income inequality is in line with the literature [44–46]. Only the Pietra index reacts to changes in *MPS* by definition. However, the increase in *MPS* in reaction to mean income growth is rather robust across specifications and data sources (LIS survey data and national accounts), in particular taking the relatively small sample size into account. Moreover, our panel regression results are robust to using random effects instead of fixed effects. As a further robustness check, we experiment with nonlinear specifications by including higher-order terms, which turn out to be statistically insignificant.

We draw three conclusions from the empirical analysis: (i) The growing mean incomes of official statistics have become less representative of the middle of the income distribution, as shown by the increase in *MPS*. Middle income earners are failing to keep up with rising mean incomes, as fits the theory on wage polarization. (ii) Increases in *MIS* are primarily associated with increasing *MPS*, and rising mean incomes did not lead to significant increases in *MIS* after the effects of *MPS* are controlled for. By contrast, the panel regressions suggest that growth has moved hand in hand with increases in *MPS*. (iii) Changes in *MPS* and *MIS* typically go into the same direction, with *MIS* changing more slowly than *MPS*.

### 3.2 Grouped data: Parametric functions and *MPS* over time

Can the parametric functions used for grouped data also capture the empirical characteristics of *MPS* and *MIS* which we have now analyzed? Let us mimic a setting in which only grouped data is available and use 5-percentile shares from our LIS data set. We fit the eight LCs from Table 3 to these twenty data points for each country and year. An often-used measure of fit criterion for parametric LCs is the mean squared error (MSE) at the 20 percentile points [27, 47]. As an alternative performance criterion, we compute the difference between the *MPS* (or respectively *MPS* + *MIS*) implied by the functional forms and their empirical values. This is in the vein of Chotikapanich [28] who compares functional forms based on the difference between the implied and empirical Gini coefficients. This allows us to answer the question if the parametric forms that perform best at the percentile points also capture *MPS* and *MIS* most accurately. Table 7 shows that the answer is no. The Kakwani LC achieves the closest fit at the percentile points for 139 out of all 175 country-year distributions, but the Rohde and Wang-Smyth LCs often imply *MPS* values which come closer to the empirical ones. This also holds when we look at the implied *MPS* and *MIS* together.

**Table 7:** Performance of functional forms.

Lowest MSE at	Pareto	Weibull	Choti.	Rohde	Logn.	Wang/S.	Vi./A.	Kakwani
20 Percentile Points					2		34	139
Implied *MPS*	1	11	15	47	17	56	12	16
Implied *MPS* + *MIS*	1	9	29	46	3	55	29	3

*Notes*: For each parametric form, the table lists the number of distributions (out of 175) for which the function has the lowest MSE, either at the 20 percentile points, *MPS* or *MPS* + *MIS*. See Table 3 for the implied *MPS* and *MIS* as well as abbreviations.

**Fig 3:**
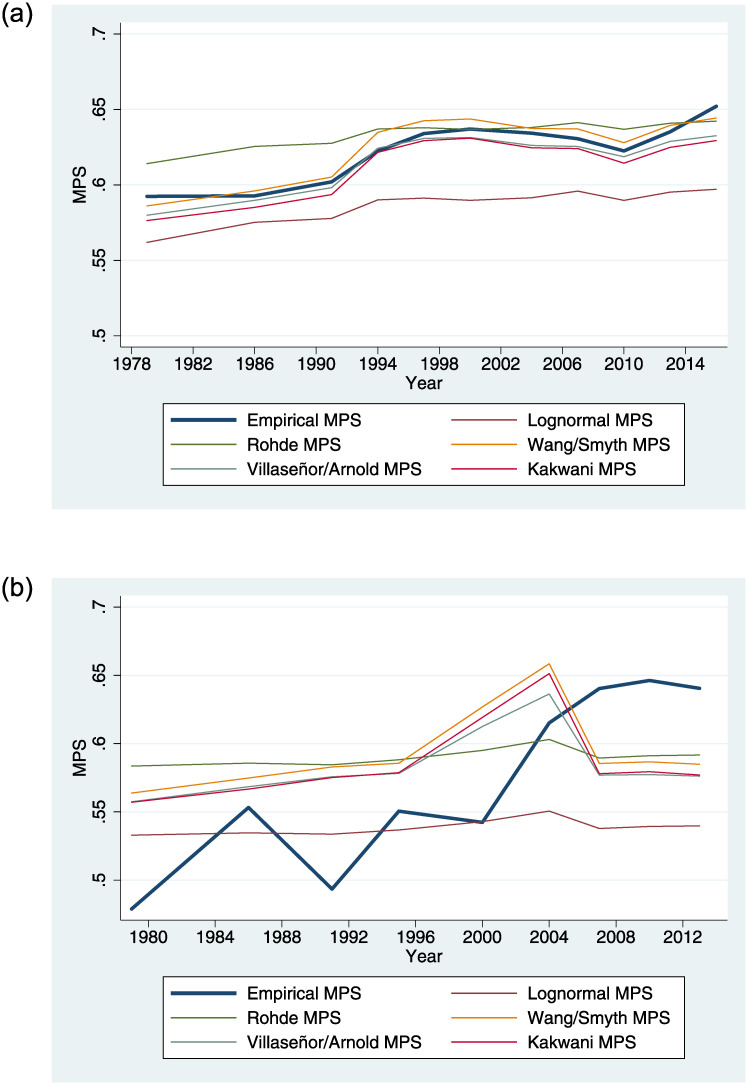
Empirical and implied MPS by parametric forms.

While this analysis was static, let us now examine how accurately the empirical evolution of a country’s *MPS* over time can be traced by the parametric functions. Here we find mixed results. In countries where *MPS*, Gini, and skewness are strongly correlated, such as the U.S., the empirical and implied *MPS* are very close (Fig 3a). By contrast, the rising *MPS* in low-inequality Norway cannot be captured well by the functional forms (Fig 3b). Obviously, the structural links between inequality and *MPS* imposed by the limited degrees of freedom can come at a disadvantage, when both move into opposite directions. Researchers working with grouped data should be aware of the choice of functional forms. Only if the implied *MPS* comes close to the empirical one can it be an appropriate measure for the inclusiveness of economic growth.

## 4 Conclusion

Rising mean incomes are a hallmark of national accounts in most countries and are generally seen as welfare improvements. Here we link mean income to two simple and intuitive statistics based on the Lorenz curve. The below-mean-income population share, *MPS*, captures how many individuals have an income below the national mean. Looking at its changes over time reflects to what extent the middle of the distribution benefits from economic growth, as *MPS* only changes when individuals move above or below the mean-income threshold. *MPS* is supplemented by its associated income share *MIS*, which comprises the percentage of total income accruing to all earners below the mean. We characterize the two statistics analytically and show how they can reflect the inclusiveness of rising mean incomes for earners at different parts of the distribution.

Our empirical analysis with 16 high- and middle-income countries shows that rising mean incomes have generally not favored middle income earners, while the combined relative welfare effects on all individuals below the mean vary across countries. Other distributional measures and inequality indices are unable to yield these findings.

*MPS* and *MIS* are very useful in tracing the distributional effects of growth across countries and time precisely thanks to their simplicity. They are insightful indices for evaluating growth inclusiveness for policymakers and can supplement official statistics by setting the distributional effects of rising mean incomes into perspective. Being very simple measures, *MPS* and *MIS* obviously have some shortcomings. Due to its particular cut-off point, *MPS* does not include middle income earners which are close to but above the mean. Also, these measures only use data from the income distribution rather than any information about the labor market or public policy. Hence, they cannot determine the root causes of distributional inequality. But they open a door to related research by drawing attention to the phenomenon of the mixed distributional welfare effects of rising mean incomes.

## Supporting information

S1 File This file contains the proofs of Theorem 1, which are the reactions of *MPS* and *MIS* to mean income growth. (PDF)

S2 File This file provides additional information on the data used in the empirical analysis as well as summary statistics and additional estimation results. (PDF)
