# Supplementary material for: Rising mean incomes for whom?
Source: PLoS One. 2020 Dec 16;15(12):e0242803. doi: 10.1371/journal.pone.0242803 (PMC7743992; doi:10.1371/journal.pone.0242803)
Supplement: S1 File — (PDF) [file pone.0242803.s001.pdf]

Appendix A Supporting Material: Proofs

A.1 Proof of Theorem 1 (Reaction of MPS to income growth)

Proof. Consider a population at time $t = 1$ with ordered incomes $y_{i,1}$, $i = 1, ..., N$, and mean income μ_1. We make use of Definition 1, $MPS_1 = F(\mu_1) = \frac{1}{N}\sum_{i=1}^{N}\mathcal{I}_{y_{i,1}\leq\mu_1}$, to derive the reactions of MPS in the five growth scenarios laid out in Theorem 1:

(a) **Uniform relative growth:** All incomes rise by the same factor $c > 1$:

$$MPS_2 = \frac{1}{N}\sum_{i=1}^{N}\mathcal{I}_{c\cdot y_{i,1}\leq c\cdot\mu_1} = \frac{1}{N}\sum_{i=1}^{N}\mathcal{I}_{y_{i,1}\leq\mu_1} = MPS_1 \tag{S-1}$$

(b) **Uniform absolute growth:** All incomes rise by the same absolute value a:

$$MPS_2 = \frac{1}{N}\sum_{i=1}^{N}\mathcal{I}_{y_{i,1}+a\leq\mu_1+a} = \frac{1}{N}\sum_{i=1}^{N}\mathcal{I}_{y_{i,1}\leq\mu_1} = MPS_1 \tag{S-2}$$

(c) **Top income growth:** The share p of top incomes grows by a factor $c > 1$, where $y_{[(1-p)N],1} > \mu_1$ implies that only incomes above the μ_1 are affected.

$$MPS_2 = \underbrace{\frac{1}{N}\sum_{i=1}^{[(1-p)N]}\mathcal{I}_{\cdot y_{i,1}\leq\mu_2}}_{\geq\frac{1}{N}\sum_{i=1}^{[(1-p)N]}\mathcal{I}_{y_{i,1}\leq\mu_1}} + \underbrace{\frac{1}{N}\sum_{i=[(1-p)N]+1}^{N}\mathcal{I}_{c\cdot y_{i,1}\leq\mu_2}}_{=0} \geq MPS_1 \tag{S-3}$$

or alternatively

$$MPS_2 = \underbrace{\frac{1}{N}\sum_{i=1}^{N}\mathcal{I}_{y_{i,1}\leq\mu_1}}_{MPS_1} + \underbrace{\frac{1}{N}\sum_{i=[N\cdot MPS_1]+1}^{[(1-p)\cdot N]}\mathcal{I}_{\mu_1\leq y_2\leq\mu_2}}_{\geq 0} \geq MPS_1, \tag{S-4}$$

with $[y]$ denoting the integer part of the number y. In (S-3), the first term captures that the incomes of some unaffected individuals just above μ_1 might fall below μ_2 because $\mu_2 > \mu_1$. The second term is zero because all top incomes affected by the growth will end up above μ_2 as $\mu_1 < \mu_2 < c \cdot \mu_1$.

(d) **Bottom income growth:** The share p of bottom incomes grow by a factor $c > 1$, where $y_{[pN],2} < \mu_1$ implies that all affected incomes remain below μ_1.

$$MPS_2 = \underbrace{\frac{1}{N}\sum_{i=1}^{[p\cdot N]} \mathcal{I}_{y_{i,2}\leq\mu_1}}_{=p \text{ as before}} + \underbrace{\frac{1}{N}\sum_{i=[p\cdot N]+1}^{N} \mathcal{I}_{y_{i,1}\leq\mu_2}}_{\geq\frac{1}{N}\sum_{i=[p\cdot N]+1}^{N} \mathcal{I}_{y_{i,1}\leq\mu_1}} \geq MPS_1 \tag{S-5}$$

In (S-5), the first term remains unchanged because the incomes of the affected individuals remain below μ_1. The second term captures incomes unaffected by the change, including those middle income earners just above μ_1, which potentially fall below μ_2 because $\mu_2 > \mu_1$.

(e) **Middle income growth:** Incomes around μ_1 between the lower bound lb and the upper bound ub grow by $c > 1$, where $y_{[1+ub\cdot N],1} > \mu_2$ implies that unaffected richer individuals stay above the new mean $\mu_2 > \mu_1$:

$$MPS_2 = \frac{1}{N}\underbrace{\sum_{i=1}^{[lb\cdot N]-1} \mathcal{I}_{y_{i,1}\leq\mu_2}}_{=[lb\cdot N]-1 \text{ as before}} + \frac{1}{N}\underbrace{\sum_{i=[lb\cdot N]}^{[ub\cdot N]} \mathcal{I}_{c\cdot y_{i,1}\leq\mu_2}}_{\leq\sum_{i=[lb\cdot N]}^{[ub\cdot N]} \mathcal{I}_{\cdot y_{i,1}\leq\mu_1}} + \frac{1}{N}\underbrace{\sum_{i=[ub\cdot N]+1}^{N} \mathcal{I}_{y_{i,1}\leq\mu_2}}_{=0 \text{ as before}} \leq MPS_1 \tag{S-6}$$

In (S-6), the first term is unchanged because unaffected poorer individuals below μ_1 are also below μ_2. The second term captures that among the affected middle income households which were below μ_1, some may jump across the mean, which increases by less than c, while their incomes increase by c. The third term captures that unaffected richer individuals stay above μ_2. This completes the proof of Theorem 1.

□

A.2 Proof of Table 1, Reaction of MIS to income growth

Proof. We proceed analogously to the previous proof and use the definition (2) of MIS

$$MIS_1 = \frac{\sum_{i=1}^{N} y_{i,1}\cdot\mathcal{I}_{y_{i,1}\leq\mu_1}}{\sum_{i=1}^{N} y_{i,1}} = \frac{\sum_{i=1}^{[MPS_1\cdot N]} y_{i,1}}{\sum_{i=1}^{N} y_{i,1}} = MPS_1\cdot\frac{\mu_{sub,1}}{\mu_1}, \tag{S-7}$$

where $\mu_{sub,1} = \frac{1}{MPS_1\cdot N}\sum_{i=1}^{N} y_{i,1}\cdot\mathcal{I}_{y_{i,1}\leq\mu_1}$ is the mean of all incomes below the mean μ_1.

(a) **Uniform relative growth:** All incomes rise by the same factor $c > 1$:

$$MIS_2 = \frac{\sum_{i=1}^{N} c \cdot y_{i,1} \cdot \mathcal{I}_{c \cdot y_{i,1} \leq c \cdot \mu_1}}{\sum_{i=1}^{N} c \cdot y_{i,1}} = \frac{\sum_{i=1}^{N} y_{i,1} \cdot \mathcal{I}_{y_{i,1} \leq \mu_1}}{\sum_{i=1}^{N} y_{i,1}} = MIS_1 \tag{S-8}$$

(b) **Uniform absolute growth:** All incomes rise by the same absolute value $a > 0$:

$$MIS_2 = \frac{\sum_{i=1}^{N} (y_{i,1} + a) \cdot \mathcal{I}_{y_{i,1}+a \leq \mu_1 + a}}{\sum_{i=1}^{N} y_{i,1} + Na} = MPS_1 \cdot \frac{\mu_{sub,1} + a}{\mu_1 + a} > MIS_1 \tag{S-9}$$

Note that $\mu_{sub,1} < \mu_1$.

(c) **Top income growth:** The share p of top incomes grows by a factor $c > 1$:

$$MIS_2 = \frac{\sum_{i=1}^{[MPS_1 \cdot N]} y_{i,1} + \sum_{i=[MPS_1 \cdot N]+1}^{[MPS_2 \cdot N]} y_{i,1}}{\mu_1 \cdot N + \sum_{i=[(1-p)N]}^{N} (c-1) \cdot y_{i,1}} \lesseqgtr MIS_1 \tag{S-10}$$

The denominator in (S-10) increases due to growth at the top. The first term in the numerator stays constant because the incomes of individuals below MPS_1 have not changed. But the second term is nonnegative as $MPS_2 > MPS_1$; it contributes the incomes of those individuals who have fallen below the mean. If this term is small (large), the numerator will increase by less (more) than the denominator and MIS will decrease (increase).

(d) **Bottom income growth:** The share p of bottom incomes grows by a factor $c > 1$:

$$MIS_2 = \frac{\sum_{i=1}^{[p \cdot N]} c \cdot y_{i,1} + \sum_{i=[p \cdot N]+1}^{[MPS_1 \cdot N]} y_{i,1} + \sum_{i=[MPS_1 \cdot N]+1}^{[MPS_2 \cdot N]} y_{i,1}}{\mu_1 \cdot N + \sum_{i=1}^{[p \cdot N]} (c-1) \cdot y_{i,1}} > MIS_1 \tag{S-11}$$

Both the numerator and the denominator in (S-11) increase, but the numerator increases by at least as much as the denominator, so that the ratio increases. The point is that all bottom incomes that go up by c are contained in the denominator as well as in the first term of the numerator. The second term in the numerator is the same as before (below-mean incomes unaffected), while the third term is nonnegative, contributing the incomes of those that fall below μ_2 as $MPS_2 > MPS_1$.

(e) **Middle income growth:** Incomes around μ_1 between the lower bound lb and the upper bound ub grow by $c > 1$:

$$MIS_2 = \frac{\sum_{i=1}^{[lb \cdot N-1]} y_{i,1} + \sum_{i=[lb \cdot N]}^{[ub \cdot N]} c \cdot y_{i,1} \mathcal{I}_{c \cdot y_{i,1} \leq \mu_2} + \sum_{i=[ub \cdot N]+1}^{[MPS_2 \cdot N]} y_{i,1} \mathcal{I}_{y_{i,1} \leq \mu_2}}{\mu_1 \cdot N + \sum_{i=[lb \cdot N]}^{[ub \cdot N]} (c-1) \cdot y_{i,1}} \lesseqgtr MIS_1 \quad \text{(S-12)}$$

While the denominator in (S-12) increases, the numerator can decrease or increase (by either less or more than the denominator) so that the overall effect is ambiguous. Note that (S-12) holds irrespective of $[MPS_2 \cdot N] > [ub \cdot N] + 1$ or $[lb \cdot N] \leq [MPS_2 \cdot N] \leq [ub \cdot N]$, in which case the third sum of the numerator is zero by definition. The point is that middle incomes which grow by c increase the numerator, but at the same time the weakly decreasing MPS implies that there are potentially fewer middle income earners below μ_2 to be included. These two effects point into opposite directions.
