# Supplementary material for: Rising mean incomes for whom?
Source: PLoS One. 2020 Dec 16;15(12):e0242803. doi: 10.1371/journal.pone.0242803 (PMC7743992; doi:10.1371/journal.pone.0242803)
Supplement: S2 File — (PDF) [file pone.0242803.s002.pdf]

Appendix B Supporting Material: Additional Statistics of the Empirical Analysis

This section provides supplementary information on the data used in the empirical analysis. It discusses statistics and estimation results. The data used is harmonized cross-country micro-level income data from the Luxembourg Income Study (LIS). Because our focus is on changes in MPS, MIS, and other statistics over time, we only use those countries that report income data for all waves at least between the 2nd (around 1985) and the 9th wave (around 2014). These are 16 high- and middle-income countries.

Table S-1 shows the countries and years used in our analysis. The number of observation years ranges between 8 (Denmark, Finland) and 26 (Germany). But thanks to the wave availability restriction imposed, they all span the period from the 1980s to the 2010s, allowing us to analyze the development over these decades.

Table S-2 to Table S-17 show MPS, $MIS = L(MPS)$ and the Gini coefficient computed for all available years per country. We do this both for total household income and disposable household income. In both cases, equivalized income is used, hence, we divide household income by the square root of the number of household members.

Following the literature, we also weight observations by the number of household members times household weights. There are a couple of countries which show marked increases in both MPS and the Gini coefficient, such as Germany, the US, and Australia. But in some other countries, such as Norway, Denmark, and Canada, MPS increased even though the Gini coefficient stayed relatively constant. In the Netherlands, MPS even decreased, a development that is not visible based on the Gini coefficient. MIS does not show pronounced changes over time in many countries. If MPS rises and MIS stays constant, such as in the US, Germany, and Finland, there are more households below the mean but their share of total income has not increased, indicating that they are relatively worse off. The tables show that the Gini coefficient based on disposable income is typically considerably lower than based on total household income, as the state redistributes from the top to the bottom. As discussed in the article, the differences between total and disposable income are only marginal for MPS, because middle-class households around the mean are often not affected as strongly by redistribution.

Table S-18 provides the growth rate of mean income g_{μ}, the growth rate of mean income for all individuals below the mean, $g_{sub\mu}$, as well as distributional metrics between the first and last available year. We can see that the group-specific mean income of individuals below national mean increased more than national mean income for both gross and disposable incomes in three countries (Denmark, Mexico, and Poland), while both MIS and MPS increased in Denmark and Poland, and decreased in Mexico. So individuals below mean income were relatively better off in the three countries because their MIS increased more than MPS did. But in the other 13 countries MPS always increased more than MIS did, making individuals at the middle and the bottom of the distribution worse off.

Table S-19 is based on the summary statistics of the 5-percentile income shares of each country. We find that the largest variation always happened to the top 5% income share, and the second largest happened to either the bottom or the second-highest (top 19th) 5% income share. We also look at the summary statistics of the interval percentiles between MIS and the bottom 6 decile income shares including MIS itself, and find that the largest variation happened to either MIS or the interval percentile between MIS and the bottom 60% income share. The second largest variation happened to the interval percentiles between MIS and either the bottom 5% income share or the bottom 50% income share.

The following three tables provide robustness checks to the panel data analysis in the paper. Table S-20 reruns the panel regressions of changes in MPS and MIS on mean income growth, both contemporaneously and with a lag. The lag is statistically

insignificant in most specification, yielding no evidence for a delayed impact of growth on MPS and MIS. The main coefficients tend to change very little with the inclusion of the lags.

Table S-21 and Table S-22 rerun the panel regressions including a country group dummy. It is remarkable that the country group dummies capture a lot of variation otherwise contained in the country fixed effects, hinting to the importance of regulatory, welfare and labor market regimes that distinguish the country groups.

While the paper used MPS and MIS as the dependent variables, Table S-23 considers the impact of mean income changes on the Gini coefficient, the skewness and the Pietra index instead. Most of the results are insignificant when the Gini coefficient and the skewness are used. The Pietra index shows similar reactions as MPS and MIS, which makes sense as they are components of the Pietra index.

Table S-24 to Table S-27 show how well the development of MPS based on disposable income can be approximated by eight different parametric forms. For each country and year, 5-percentile income shares are used (5%, 10%, 15%...), mimicking the grouped-data available in many cross-country data sets. The eight parametric LCs from Table 2 in the paper (Lognormal, Chotikapanich, Pareto, Rohde, Weibull, Wang/Smyth, Villaseñor/Arnold and Kakwani) are fitted to these 20 data points. Based on their mean squared error minimizing parameter(s), these forms imply a particular MPS value, which one can compute with the formulas from Table 3 in the paper. Here we present the MPS implied by all the parametric forms, together with the empirical MPS, highlighting in bold which form comes closest to the empirical value. We can see that there is a lot of heterogeneity between the parametric functions in terms of their ability to capture the empirical MPS. The Pareto LC typically implies MPS values which are too high, while the Lognormal-implied ones often lie below the empirical values. Many of the other forms come quite close and in particular the Rohde and Wang/Smyth LCs perform best in capturing the evolution of MPS in many countries. As described in the article, the Kakwani LC clearly dominates the other forms in terms of fit at the 20 percentile points, but we see a different picture when it comes to representing MPS. Researchers choosing parametric LCs should take care.

Table S-1: Available LIS Microdata Across Countries and Years

Year	AU	CA	DE	DK	ES	FI	IL	IT	LU	MX	NL	NO	PL	TW	UK	US
1978			X													
1979							X					X			X	X
1980					X											
1981	X	X	X											X		
1982																
1983			X								X					
1984			X							X						
1985	X				X				X							
1986							X	X				X	X	X	X	X
1987		X	X	X		X		X			X					
1988																
1989	X		X					X		X						
1990					X						X					
1991		X	X			X		X	X			X		X	X	X
1992				X			X			X			X			
1993								X			X					
1994		X	X						X	X					X	X
1995	X		X	X	X	X		X				X	X	X	X	
1996										X						
1997		X					X		X					X		X
1998		X	X					X		X						
1999											X		X		X	
2000		X	X	X	X	X		X	X	X		X		X		X
2001	X		X					X								
2002			X							X						
2003	X		X													
2004	X	X	X	X	X	X		X	X	X	X	X	X		X	X
2005			X				X							X		
2006			X													
2007		X	X	X	X	X	X		X		X	X	X	X	X	X
2008	X		X					X		X						
2009			X													
2010	X	X	X	X	X	X	X	X	X	X	X	X	X	X	X	X
2011			X													
2012			X				X			X						
2013		X	X	X	X	X			X		X	X	X	X	X	X
2014	X		X				X	X								
2015			X													
2016							X						X	X		X

Notes: The table shows the years for which country microdata is available in the LIS database and has been used for the empirical analysis.

Table S-2: Statistics for Australia (AU)

Year	Total Income			Disposable Income		
	MPS	MIS	Gini	MPS	MIS	Gini
1981	0.5952	0.3564	0.3266	0.5835	0.3782	0.2807
1985	0.6045	0.3557	0.3464	0.5811	0.3714	0.2915
1989	0.6159	0.3560	0.3534	0.5992	0.3769	0.3025
1995	0.6342	0.3577	0.3539	0.6163	0.3768	0.3043
2001	0.6399	0.3588	0.3636	0.6241	0.3800	0.3141
2003	0.6439	0.3651	0.3592	0.6307	0.3888	0.3099
2004	0.6398	0.3711	0.3574	0.6325	0.3971	0.3131
2008	0.6649	0.3817	0.3788	0.6540	0.4052	0.3332
2010	0.7176	0.4111	0.3696	0.7100	0.4351	0.3307
2014	0.6878	0.3908	0.3899	0.6676	0.4086	0.3385

Notes: The statistics are based on LIS equivalized household income microdata. Equivalized income is obtained by dividing by the square root of household members. Observations are weighted by the number of household members times household weights.

Table S-3: Statistics for Canada (CA)

Year	Total Income			Disposable Income		
	MPS	MIS	Gini	MPS	MIS	Gini
1981	0.6257	0.3855	0.3119	0.6159	0.3996	0.2831
1987	0.6665	0.4116	0.3175	0.6571	0.4306	0.2821
1991	0.6553	0.4009	0.3218	0.6450	0.4232	0.2806
1994	0.6392	0.3870	0.3269	0.6285	0.4108	0.2836
1997	0.6468	0.3851	0.3331	0.6368	0.4085	0.2912
1998	0.6557	0.3813	0.3566	0.6418	0.4015	0.3109
2000	0.6582	0.3838	0.3593	0.6497	0.4091	0.3160
2004	0.6430	0.3745	0.3592	0.6370	0.3979	0.3191
2007	0.6438	0.3788	0.3583	0.6356	0.4012	0.3177
2010	0.6277	0.3689	0.3579	0.6184	0.3895	0.3173
2013	0.6283	0.3619	0.3577	0.6248	0.3857	0.3208

Notes: The statistics are based on LIS equivalized household income microdata. Equivalized income is obtained by dividing by the square root of household members. Observations are weighted by the number of household members times household weights.

Table S-4: Statistics for Germany (DE)

Year	Total Income MPS	Total Income MIS	Total Income Gini	Disposable Income MPS	Disposable Income MIS	Disposable Income Gini
1978	0.5815	0.3887	0.2918	0.5630	0.3926	0.2635
1981	0.6018	0.4007	0.2732	0.5985	0.4219	0.2439
1983	0.5426	0.3623	0.2855	0.5310	0.3696	0.2605
1984	0.5981	0.3795	0.3052	0.5959	0.4089	0.2540
1987	0.5941	0.3825	0.3002	0.6004	0.4200	0.2509
1989	0.6076	0.3924	0.3077	0.6089	0.4248	0.2556
1991	0.6324	0.3995	0.3106	0.6348	0.4299	0.2670
1994	0.6227	0.3932	0.3131	0.6303	0.4349	0.2621
1995	0.6243	0.3914	0.3146	0.6264	0.4326	0.2573
1998	0.6363	0.4022	0.3117	0.6262	0.4369	0.2530
2000	0.6311	0.3972	0.3181	0.6196	0.4304	0.2586
2001	0.5690	0.3565	0.3345	0.5540	0.3824	0.2723
2002	0.5687	0.3518	0.3317	0.5551	0.3812	0.2703
2003	0.5738	0.3533	0.3372	0.5550	0.3794	0.2715
2004	0.5803	0.3600	0.3361	0.5680	0.3902	0.2757
2005	0.5930	0.3593	0.3504	0.5829	0.3916	0.2930
2006	0.5926	0.3606	0.3437	0.5837	0.3933	0.2875
2007	0.6000	0.3634	0.3461	0.5906	0.3963	0.2899
2008	0.6023	0.3668	0.3441	0.5936	0.3981	0.2891
2009	0.6553	0.3979	0.3385	0.6481	0.4341	0.2848
2010	0.6593	0.3976	0.3390	0.6472	0.4269	0.2854
2011	0.6637	0.4027	0.3422	0.6517	0.4339	0.2861
2012	0.6609	0.3974	0.3455	0.6542	0.4331	0.2890
2013	0.6540	0.3923	0.3492	0.6456	0.4265	0.2916
2014	0.6513	0.3901	0.3441	0.6459	0.4251	0.2894
2015	0.6434	0.3813	0.3528	0.6340	0.4148	0.2958

Notes: The statistics are based on LIS equivalized household income microdata. Equivalized income is obtained by dividing by the square root of household members. Observations are weighted by the number of household members times household weights.

Table S-5: Statistics for Denmark (DK)

	Total Income			Disposable Income		
Year	MPS	MIS	Gini	MPS	MIS	Gini
1987	0.6091	0.3876	0.2798	0.6292	0.4261	0.2521
1992	0.6159	0.3884	0.2828	0.6290	0.4347	0.2371
1995	0.6380	0.4320	0.2621	0.6309	0.4531	0.2207
2000	0.6382	0.4260	0.2714	0.6258	0.4461	0.2248
2004	0.6391	0.4256	0.2709	0.6253	0.4421	0.2284
2007	0.6452	0.4244	0.2829	0.6315	0.4412	0.2391
2010	0.6602	0.4291	0.2925	0.6460	0.4422	0.2518
2013	0.6641	0.4368	0.2888	0.6546	0.4550	0.2505

Notes: The statistics are based on LIS equivalized household income microdata. Equivalized income is obtained by dividing by the square root of household members. Observations are weighted by the number of household members times household weights.

Table S-6: Statistics for Spain (ES)

	Total Income			Disposable Income		
Year	MPS	MIS	Gini	MPS	MIS	Gini
1980	0.6315	0.3987	0.3203	0.6315	0.3987	0.3203
1985	0.6227	0.4030	0.3149	0.6227	0.4030	0.3149
1990	0.6338	0.4091	0.3040	0.6338	0.4091	0.3040
1995	0.6376	0.3766	0.3513	0.6376	0.3766	0.3513
2000	0.6503	0.3958	0.3356	0.6503	0.3958	0.3356
2004	0.6372	0.3937	0.3177	0.6375	0.3941	0.3162
2007	0.6476	0.3920	0.3265	0.6319	0.3940	0.3036
2010	0.6315	0.3626	0.3466	0.6166	0.3649	0.3274
2013	0.6187	0.3458	0.3771	0.5944	0.3504	0.3405

Notes: The statistics are based on LIS equivalized household income microdata. Equivalized income is obtained by dividing by the square root of household members. Observations are weighted by the number of household members times household weights.

Table S-7: Statistics for Finland (FI)

Year	Total Income MPS	Total Income MIS	Total Income Gini	Disposable Income MPS	Disposable Income MIS	Disposable Income Gini
1987	0.5039	0.3432	0.2591	0.4876	0.3589	0.2069
1991	0.5421	0.3722	0.2571	0.5266	0.3875	0.2088
1995	0.5452	0.3700	0.2718	0.5386	0.3988	0.2171
2000	0.5183	0.3361	0.3034	0.5033	0.3522	0.2558
2004	0.5826	0.3769	0.3109	0.5703	0.3965	0.2658
2007	0.5608	0.3608	0.3073	0.5483	0.3770	0.2665
2010	0.5752	0.3693	0.3043	0.5620	0.3865	0.2632
2013	0.5578	0.3585	0.3057	0.5429	0.3756	0.2609

Notes: The statistics are based on LIS equivalized household income microdata. Equivalized income is obtained by dividing by the square root of household members. Observations are weighted by the number of household members times household weights.

Table S-8: Statistics for Israel (IL)

Year	Total Income MPS	Total Income MIS	Total Income Gini	Disposable Income MPS	Disposable Income MIS	Disposable Income Gini
1979	0.5605	0.3232	0.3585	0.5253	0.3320	0.3038
1986	0.6062	0.3475	0.3772	0.5629	0.3559	0.3098
1992	0.6253	0.3654	0.3631	0.5825	0.3702	0.3055
1997	0.6335	0.3511	0.3971	0.5883	0.3549	0.3371
2001	0.6339	0.3392	0.4136	0.5823	0.3404	0.3486
2005	0.6234	0.3278	0.4220	0.5832	0.3274	0.3754
2007	0.6270	0.3305	0.4170	0.5851	0.3267	0.3677
2010	0.6278	0.3284	0.4290	0.5864	0.3218	0.3836
2012	0.6251	0.3340	0.4105	0.5900	0.3306	0.3697
2014	0.6392	0.3441	0.4015	0.6027	0.3379	0.3586
2016	0.6275	0.3438	0.3915	0.5854	0.3383	0.3451

Notes: The statistics are based on LIS equivalized household income microdata. Equivalized income is obtained by dividing by the square root of household members. Observations are weighted by the number of household members times household weights.

Table S-9: Statistics for Italy (IT)

Year	MPS	MIS	Gini
1986	0.5944	0.3778	0.3093
1987	0.5533	0.3421	0.3327
1989	0.6036	0.3888	0.3033
1991	0.6172	0.4009	0.2910
1993	0.6155	0.3673	0.3406
1995	0.6125	0.3685	0.3369
1998	0.6039	0.3653	0.3422
2000	0.6024	0.3711	0.3291
2004	0.6206	0.3850	0.3339
2008	0.6162	0.3856	0.3219
2010	0.5946	0.3687	0.3196
2014	0.5778	0.3538	0.3204

Notes: The statistics are based on LIS equivalized household income microdata. Equivalized income is obtained by dividing by the square root of household members. Observations are weighted by the number of household members times household weights. The data for Italy does not differentiate between total and disposable income.

Table S-10: Statistics for Luxembourg (LU)

	Total Income			Disposable Income		
Year	MPS	MIS	Gini	MPS	MIS	Gini
1985	0.5348	0.3825	0.2357	0.5348	0.3825	0.2357
1991	0.5933	0.4214	0.2388	0.5933	0.4214	0.2388
1994	0.6045	0.4312	0.2354	0.6045	0.4312	0.2354
1997	0.5843	0.4045	0.2610	0.5843	0.4045	0.2610
2000	0.5905	0.4050	0.2621	0.5905	0.4050	0.2621
2004	0.6204	0.4029	0.3065	0.6129	0.4225	0.2696
2007	0.6434	0.4112	0.3101	0.6383	0.4321	0.2764
2010	0.6073	0.3880	0.3047	0.5917	0.3968	0.2702
2013	0.5824	0.3646	0.3172	0.5670	0.3734	0.2793

Notes: The statistics are based on LIS equivalized household income microdata. Equivalized income is obtained by dividing by the square root of household members. Observations are weighted by the number of household members times household weights.

Table S-11: Statistics for Mexico (MX)

Year	MPS	MIS	Gini
1984	0.6347	0.3350	0.4328
1989	0.6672	0.3435	0.4762
1992	0.7334	0.3608	0.5005
1994	0.7401	0.3582	0.5052
1996	0.7139	0.3528	0.4913
1998	0.7042	0.3351	0.5047
2000	0.7208	0.3473	0.4992
2002	0.7087	0.3556	0.4749
2004	0.6479	0.3276	0.4710
2008	0.6640	0.3227	0.4870
2010	0.6791	0.3408	0.4613
2012	0.7026	0.3464	0.4672

Notes: The statistics are based on LIS equivalized household income microdata. Equivalized income is obtained by dividing by the square root of household members. Observations are weighted by the number of household members times household weights. The data for Mexico does not differentiate between total and disposable income.

Table S-12: Statistics for the Netherlands (NL)

	Total Income			Disposable Income		
Year	MPS	MIS	Gini	MPS	MIS	Gini
1983	0.6219	0.4094	0.2955	0.6127	0.4292	0.2517
1987	0.6294	0.4211	0.2815	0.6070	0.4338	0.2280
1990	0.6016	0.3972	0.2869	0.5984	0.4076	0.2633
1993	0.5804	0.3698	0.2989	0.5556	0.3667	0.2558
1999	0.5860	0.3927	0.2742	0.5726	0.4117	0.2303
2004	0.5777	0.3712	0.3068	0.5615	0.3845	0.2636
2007	0.5618	0.3525	0.3254	0.5688	0.3930	0.2744
2010	0.5386	0.3444	0.3045	0.5305	0.3671	0.2544
2013	0.5322	0.3367	0.3150	0.5291	0.3653	0.2615

Notes: The statistics are based on LIS equivalized household income microdata. Equivalized income is obtained by dividing by the square root of household members. Observations are weighted by the number of household members times household weights.

Table S-13: Statistics for Norway (NO)

	Total Income			Disposable Income		
Year	MPS	MIS	Gini	MPS	MIS	Gini
1979	0.4837	0.3193	0.2738	0.4789	0.3428	0.2234
1986	0.5468	0.3680	0.2645	0.5532	0.3950	0.2337
1991	0.5032	0.3393	0.2714	0.4935	0.3558	0.2315
1995	0.5498	0.3621	0.2820	0.5505	0.3899	0.2414
2000	0.5354	0.3552	0.2975	0.5423	0.3841	0.2591
2004	0.6070	0.4016	0.3110	0.6152	0.4319	0.2816
2007	0.6532	0.4346	0.2886	0.6403	0.4531	0.2449
2010	0.6603	0.4373	0.2929	0.6462	0.4547	0.2480
2013	0.6542	0.4301	0.2951	0.6405	0.4485	0.2500

Notes: The statistics are based on LIS equivalized household income microdata. Equivalized income is obtained by dividing by the square root of household members. Observations are weighted by the number of household members times household weights.

Table S-14: Statistics for Poland (PL)

	Total Income			Disposable Income		
Year	MPS	MIS	Gini	MPS	MIS	Gini
1986	0.6151	0.4096	0.2708	0.6151	0.4096	0.2708
1992	0.6417	0.4463	0.2621	0.6417	0.4463	0.2621
1995	0.5773	0.3552	0.3113	0.5853	0.3592	0.3111
1999	0.5978	0.3909	0.2930	0.6045	0.4003	0.2873
2004	0.5970	0.3734	0.3210	0.6004	0.3803	0.3156
2007	0.6377	0.4097	0.3144	0.6387	0.4119	0.3110
2010	0.6362	0.4091	0.3110	0.6365	0.4097	0.3095
2013	0.6322	0.3992	0.3169	0.6316	0.3977	0.3159
2016	0.6426	0.4255	0.2836	0.6426	0.4247	0.2833

Notes: The statistics are based on LIS equivalized household income microdata. Equivalized income is obtained by dividing by the square root of household members. Observations are weighted by the number of household members times household weights.

Table S-15: Statistics for Taiwan (TW)

Year	Total Income MPS	Total Income MIS	Total Income Gini	Disposable Income MPS	Disposable Income MIS	Disposable Income Gini
1981	0.6089	0.4196	0.2715	0.6056	0.4193	0.2672
1986	0.6272	0.4320	0.2762	0.6232	0.4317	0.2707
1991	0.6252	0.4256	0.2777	0.6224	0.4267	0.2725
1995	0.6214	0.4156	0.2884	0.6190	0.4162	0.2844
1997	0.6170	0.4106	0.2890	0.6110	0.4062	0.2874
2000	0.6202	0.4117	0.2920	0.6155	0.4094	0.2891
2005	0.6490	0.4218	0.3087	0.6453	0.4212	0.3053
2007	0.6472	0.4229	0.3044	0.6397	0.4148	0.3070
2010	0.6420	0.4159	0.3074	0.6364	0.4054	0.3166
2013	0.6530	0.4285	0.3045	0.6443	0.4187	0.3077
2016	0.6591	0.4345	0.2981	0.6519	0.4244	0.3031

Notes: The statistics are based on LIS equivalized household income microdata. Equivalized income is obtained by dividing by the square root of household members. Observations are weighted by the number of household members times household weights.

Table S-16: Statistics for the United Kingdom (UK)

Year	Total Income MPS	Total Income MIS	Total Income Gini	Disposable Income MPS	Disposable Income MIS	Disposable Income Gini
1979	0.6002	0.3789	0.2950	0.6088	0.4075	0.2652
1986	0.6214	0.3645	0.3394	0.6110	0.3828	0.2959
1991	0.6345	0.3574	0.3705	0.6344	0.3825	0.3380
1994	0.6721	0.3819	0.3725	0.6764	0.4104	0.3415
1995	0.6319	0.3489	0.3798	0.6322	0.3756	0.3429
1999	0.6675	0.3779	0.3788	0.6656	0.3948	0.3497
2004	0.6893	0.3990	0.3747	0.6945	0.4229	0.3511
2007	0.6894	0.3958	0.3735	0.6795	0.4158	0.3399
2010	0.6905	0.4029	0.3718	0.6845	0.4286	0.3348
2013	0.6876	0.4040	0.3654	0.6813	0.4261	0.3307

Notes: The statistics are based on LIS equivalized household income microdata. Equivalized income is obtained by dividing by the square root of household members. Observations are weighted by the number of household members times household weights.

Table S-17: Statistics for the United States (US)

Year	Total Income MPS	Total Income MIS	Total Income Gini	Disposable Income MPS	Disposable Income MIS	Disposable Income Gini
1979	0.6128	0.3542	0.3538	0.5924	0.3672	0.3089
1986	0.6177	0.3395	0.3836	0.5927	0.3477	0.3391
1991	0.6232	0.3411	0.3871	0.6020	0.3519	0.3449
1994	0.6444	0.3466	0.4138	0.6225	0.3589	0.3692
1997	0.6578	0.3561	0.4174	0.6340	0.3684	0.3717
2000	0.6621	0.3577	0.4201	0.6370	0.3726	0.3693
2004	0.6541	0.3514	0.4173	0.6343	0.3656	0.3727
2007	0.6465	0.3469	0.4153	0.6306	0.3571	0.3767
2010	0.6440	0.3427	0.4159	0.6225	0.3558	0.3690
2013	0.6547	0.3466	0.4244	0.6351	0.3609	0.3791
2016	0.6709	0.3531	0.4309	0.6521	0.3735	0.3825

Notes: The statistics are based on LIS equivalized household income microdata. Equivalized income is obtained by dividing by the square root of household members. Observations are weighted by the number of household members times household weights.

Table S-18: Growth Rates of Mean Incomes and Distribution Metrics

Country	*Gross income data*					*Disposable income data*				
	g_μ	$g_{sub\mu}$	g_{MPS}	g_{MIS}	g_{Gini}	g_μ	$g_{sub\mu}$	g_{MPS}	g_{MIS}	g_{Gini}
DK	0.551	0.566	0.083	0.113	0.031	0.547	0.559	0.039	0.064	-0.006
PL	0.782	0.797	0.102	0.165	-0.098	0.809	0.822	0.089	0.154	-0.098
MX	0.830	0.833	-0.053	-0.034	-0.081	0.830	0.833	-0.053	-0.034	-0.081
FI	0.231	0.226	-0.044	-0.051	-0.017	0.244	0.240	-0.050	-0.056	-0.019
IT	0.074	0.062	-0.074	-0.088	-0.042	0.074	0.062	-0.074	-0.088	-0.042
NL	0.160	0.125	-0.168	-0.216	0.062	0.147	0.134	-0.158	-0.175	0.037
ES	0.333	0.263	-0.030	-0.138	0.158	0.195	0.156	-0.073	-0.125	0.071
LU	0.189	0.159	-0.065	-0.105	0.034	0.155	0.115	-0.081	-0.132	0.035
CA	0.714	0.694	0.004	-0.065	0.128	0.700	0.685	0.014	-0.036	0.118
DE	0.204	0.102	0.096	-0.019	0.173	0.119	0.062	0.112	0.053	0.109
US	0.779	0.758	0.087	-0.003	0.179	0.777	0.758	0.091	0.017	0.192
NO	0.883	0.883	0.261	0.258	0.072	0.883	0.880	0.252	0.236	0.106
TW	0.782	0.772	0.076	0.034	0.089	0.750	0.735	0.071	0.012	0.118
AU	0.825	0.815	0.135	0.088	0.162	0.831	0.821	0.126	0.074	0.171
IL	0.871	0.864	0.107	0.060	0.084	0.889	0.879	0.103	0.019	0.120
UK	0.852	0.841	0.127	0.062	0.193	0.857	0.847	0.106	0.044	0.198

Notes: The growth of a variable is calculated between the first and last year available, where to keep the statistic units comparable, the first year of ES, FI, IT, and LU is 2004, IL 2010, MX 1994, and PL 1995. g_μ is the growth rate of national mean income, $g_{sub\mu}$ is the growth rate of mean income for individuals below mean income. The left 5 columns are calculated from gross income data, and the right 5 columns are from disposable income data.

Table S-19: Top 2 Largest Standard Deviations of Interval Income Shares

Country	CA	IL	DE	MX	US	FI	TW	UK	AU	DK	LU	NO	ES	IT	NL	PL
P5	0.001	0.004	0.002	0.003	0.001	0.001	0.003	0.003	0.002	0.003	0.003	0.002	0.003	0.002	0.005	0.006
P95	0.002	0.004	0.002	0.003	0.003	0.003	0.003	0.003	0.002	0.002	0.002	0.002	0.002	0.002	0.002	0.003
P100	0.013	0.013	0.010	0.020	0.018	0.016	0.005	0.019	0.016	0.008	0.009	0.019	0.009	0.010	0.013	0.015
MD5	0.013	0.013	0.022	0.012	0.008	0.017	0.008	0.019	0.020	0.008	0.020	0.044	0.017	0.014	0.024	0.021
MD50	0.009	0.013	0.024	0.018	0.016	0.024	0.011	0.024	0.028	0.010	0.021	0.048	0.011	0.012	0.022	0.018
MD60	0.009	0.014	0.024	0.021	0.019	0.026	0.011	0.027	0.030	0.011	0.021	0.049	0.013	0.012	0.022	0.018
MIS	0.013	0.015	0.021	0.012	0.008	0.017	0.008	0.020	0.020	0.009	0.021	0.043	0.019	0.016	0.027	0.025

Notes: P5 is bottom 5% income share, P95 is the 19th percentile (second highest), P100 is the top 5% income share; D50 is the bottom 50% income share and D60 is the bottom 60% income share. MD5=MIS-P5, MD50=MIS-D50, MD60=MIS-D60. The data is for disposable income.

Table S-20: Panel Regressions of Changes in MPS and MIS on Mean Income Growth with Lags

Panel A: Nominal growth	*Dependent variable:* ΔMPS			
growth_nom	0.006	0.006	-0.001	0.006
	(0.011)	(0.006)	(0.014)	(0.006)
lag_growth_nom	-0.001	0.003	-0.001	0.003
	(0.004)	(0.002)	(0.005)	(0.002)
ΔMIS		1.066***		1.066***
		(0.114)		(0.104)
Adjusted R^2	0.0269	0.737	0.106	0.767
Country FE	Yes	Yes	Yes	Yes
Year FE	No	No	Yes	Yes
Time Trend	Yes	Yes	Yes	Yes
Observations	158	158	158	158
Countries	16	16	16	16

Panel B: Real growth	*Dependent variable:* ΔMPS			
growth_real	0.111**	0.011	0.151 **	0.036**
	(0.048)	(0.019)	(0.062)	(0.012)
lag_growth_real	-0.008	-0.003	-0.005 **	-0.007*
	(0.006)	(0.005)	(0.009)	(0.002)
ΔMIS		1.121***		1.115***
		(0.121)		(0.116)
Adjusted R^2	0.0894	0.732	0.173	0.771
Country FE	Yes	Yes	Yes	Yes
Year FE	No	No	Yes	Yes
Time Trend	Yes	Yes	Yes	Yes
Observations	145	145	145	145
Countries	15	15	15	15

Panel C: Nominal growth	*Dependent variable:* ΔMIS			
growth_nom	-0.001	-0.004	-0.007	-0.006
	(0.008)	(0.004)	(0.013)	(0.006)
lag_growth_nom	-0.003	-0.003	-0.004	-0.003
	(0.004)	(0.002)	(0.006)	(0.003)
ΔMPS		0.686***		0.696***
		(0.066)		(0.058)
Adjusted R^2	0.0315	0.738	0.0973	0.765
Country FE	Yes	Yes	Yes	Yes
Year FE	No	No	Yes	Yes
Time Trend	Yes	Yes	Yes	Yes
Observations	158	158	158	158
Countries	16	16	16	16

Panel D: Real growth	*Dependent variable:* ΔMIS			
growth_real	0.090*	0.020	0.103*	0.005
	(0.043)	(0.020)	(0.057)	(0.024)
lag_growth_real	-0.005	0.000	0.002	0.005*
	(0.005)	(0.004)	(0.007)	(0.002)
ΔMPS		0.631***		0.650***
		(0.055)		(0.051)
Adjusted R^2	0.0744	0.728	0.0130	0.759
Country FE	Yes	Yes	Yes	Yes
Year FE	No	No	Yes	Yes
Time Trend	Yes	Yes	Yes	Yes
Observations	145	145	145	145
Countries	15	15	15	15

Notes: Standard errors are in parentheses. Compared to the main regressions in the text, these regressions include the lagged value of mean income growth. Significant at: * $p < 0.10$, ** $p < 0.05$, *** $p < 0.01$.

Table S-21: Panel Regressions of Changes in MPS and MIS on Mean Income Growth with Country Group Dummies (Part 1)

Panel A: Nominal growth	*Dependent variable: ΔMPS*			
growth_nom	0.007*	0.001	0.003	0.002
	(0.004)	(0.002)	(0.005)	(0.002)
ΔMIS		1.049***		1.052***
		(0.053)		(0.054)
anglo	0.007	0.003	0.006	0.003
	(0.005)	(0.003)	(0.005)	(0.003)
nordic	0.011**	0.001	0.012**	0.001
	(0.006)	(0.003)	(0.006)	(0.003)
med	-0.000	0.000	0.001	0.000
	(0.005)	(0.003)	(0.005)	(0.003)
Adjusted R^2	0.0434	0.730	0.110	0.721
Country FE	Yes	Yes	Yes	Yes
Year FE	No	No	Yes	Yes
Time Trend	Yes	Yes	Yes	Yes
Observations	159	159	159	159
Countries	16	16	16	16

Panel B: Real growth	*Dependent variable: ΔMPS*			
growth_real	0.085***	0.018	0.114 ***	0.031*
	(0.031)	(0.017)	(0.039)	(0.016)
ΔMIS		1.049***		1.047***
		(0.056)		(0.056)
anglo	0.006	0.003	0.006	0.003
	(0.005)	(0.003)	(0.005)	(0.003)
nordic	0.011*	0.001	0.013**	0.001
	(0.006)	(0.003)	(0.006)	(0.003)
med	0.000	0.000	0.003	0.000
	(0.005)	(0.003)	(0.005)	(0.003)
Adjusted R^2	0.0746	0.734	0.154	0.728
Country FE	Yes	Yes	Yes	Yes
Year FE	No	No	Yes	Yes
Time Trend	Yes	Yes	Yes	Yes
Observations	148	148	148	148
Countries	15	15	15	15

Panel C: Nominal growth	*Dependent variable: ΔMIS*			
growth_nom	0.06***	0.001	0.002	0.000
	(0.003)	(0.002)	(0.004)	(0.002)
ΔMPS		0.686***		0.678***
		(0.035)		(0.035)
anglo	0.003	-0.001	0.003	-0.001
	(0.004)	(0.002)	(0.004)	(0.002)
nordic	0.0009**	0.002	0.012**	0.002
	(0.005)	(0.002)	(0.005)	(0.002)
med	-0.001	-0.000	0.002	-0.000
	(0.004)	(0.002)	(0.004)	(0.002)
Adjusted R^2	0.0232	0.724	0.0702	0.719
Country FE	Yes	Yes	Yes	Yes
Year FE	No	No	Yes	Yes
Time Trend	Yes	Yes	Yes	Yes
Observations	159	159	159	159
Countries	16	16	16	16

Notes: Standard errors are in parentheses. The country groups captured by the dummy are anglo-saxon (AU, CA, UK, US), nordic (DK, FI, NO), mediterranean (ES, IL, IT), and as the reference category the diverse group of remaining countries (DE, LU, MX, NL, PL, TW). Significant at: * $p < 0.10$, ** $p < 0.05$, *** $p < 0.01$.

Table S-22: Panel Regressions of Changes in MPS and MIS on Mean Income Growth with Country Group Dummies (Part 2)

Panel D: Real growth	*Dependent variable:* ΔMIS			
growth_real	0.064**	0.006	0.071*	-0.003
	(0.025)	(0.014)	(0.032)	(0.013)
ΔMPS		0.681***		0.676***
		(0.036)		(0.036)
anglo	0.003	-0.001	0.004	-0.001
	(0.004)	(0.002)	(0.004)	(0.002)
nordic	0.0009**	0.002	0.013**	0.002
	(0.005)	(0.003)	(0.005)	(0.003)
med	0.000	0.000	0.004	-0.000
	(0.004)	(0.002)	(0.004)	(0.002)
Adjusted R^2	0.0463	0.726	0.0888	0.721
Country FE	Yes	Yes	Yes	Yes
Year FE	No	No	Yes	Yes
Time Trend	Yes	Yes	Yes	Yes
Observations	148	148	148	148
Countries	15	15	15	15

Notes: This table is the continuation of Table S-21.

Table S-23: Panel Regressions of Changes in Measures on Mean Income Growth

Panel A: Nominal Growth, time trend	*Dependent variable:*					
	$\Delta Gini$		$\Delta Skew$		$\Delta Pietra$	
growth_nom	0.003	0.004	4.328	5.457	0.000	-0.002
	(0.006)	(0.006)	(4.982)	(5.875)	(0.004)	(0.003)
ΔMPS		-0.106		-164.464		0.311***
		(0.070)		(108.702)		(0.078)
Country FE	Yes	Yes	Yes	Yes	Yes	Yes
Year FE	No	No	No	No	No	No
Time Trend	Yes	Yes	Yes	Yes	Yes	Yes
Observations	159	159	159	159	159	159
Countries	16	16	16	16	16	16

Panel B: Nominal Growth, year FE	*Dependent variable:*					
	$\Delta Gini$		$\Delta Skew$		$\Delta Pietra$	
growth_nom	0.005	0.005	7.469	7.818	-0.001	-0.002
	(0.005)	(0.005)	(5.203)	(5.098)	(0.004)	(0.003)
ΔMPS		-0.102		-173.592		0.300***
		(0.074)		(143.024)		(0.073)
Country FE	Yes	Yes	Yes	Yes	Yes	Yes
Year FE	Yes	Yes	Yes	Yes	Yes	Yes
Time Trend	No	No	No	No	No	No
Observations	159	159	159	159	159	159
Countries	16	16	16	16	16	16

Panel C: Real Growth, time trend	*Dependent variable:*					
	$\Delta Gini$		$\Delta Skew$		$\Delta Pietra$	
growth_real	-0.029	-0.019	-4.733	13.235	0.017	-0.018
	(0.029)	(0.028)	(38.882)	(43.006)	(0.017)	(0.016)
ΔMPS		-0.090		-165.738		0.322***
		(0.082)		(114.545)		(0.080)
Country FE	Yes	Yes	Yes	Yes	Yes	Yes
Year FE	No	No	No	No	No	No
Time Trend	Yes	Yes	Yes	Yes	Yes	Yes
Observations	148	148	148	148	148	148
Countries	15	15	15	15	15	15

Panel D: Real Growth, year FE	*Dependent variable:*					
	$\Delta Gini$		$\Delta Skew$		$\Delta Pietra$	
growth_real	-0.039	-0.026	-13.977	11.989	0.037**	-0.007
	(0.037)	(0.041)	(44.507)	(56.413)	(0.015)	(0.025)
ΔMPS		-0.089		-178.003		0.302***
		(0.089)		(167.223)		(0.080)
Country FE	Yes	Yes	Yes	Yes	Yes	Yes
Year FE	Yes	Yes	Yes	Yes	Yes	Yes
Time Trend	No	No	No	No	No	No
Observation	148	148	148	148	148	148
Countries	15	15	15	15	15	15

Notes: Standard errors clustered at the country level are in parentheses. Significant at: * $p < 0.10$, ** $p < 0.05$, *** $p < 0.01$.

Table S-24: Empirical MPS and the MPS Implied by the Parametric Forms (Part 1)

	MPS	Logn	Pareto	Chot.	Rohde	Weib.	Wang/S.	Vi./Arn.	Kakwani
					Australia (AU)				
1981	0.5835	0.5506	0.7254	0.5705	0.6033	0.5396	**0.5738**	0.5692	0.5641
1985	0.5811	0.5547	0.7291	0.5733	0.6074	0.5440	**0.5848**	0.5795	0.5744
1989	0.5992	0.5591	0.7328	0.5762	0.6116	0.5485	**0.5936**	0.5878	0.5824
1995	0.6163	0.5638	0.7363	0.5793	**0.6158**	0.5533	0.5907	0.5848	0.5798
2001	0.6241	0.5664	0.7384	0.5809	**0.6181**	0.5557	0.6014	0.5950	0.5900
2003	0.6307	0.5641	0.7366	0.5794	**0.6161**	0.5535	0.5967	0.5908	0.5853
2004	0.6325	0.5647	0.7374	0.5796	**0.6166**	0.5539	0.6110	0.6033	0.5991
2008	0.6540	0.5734	0.7441	0.5848	0.6240	0.5618	**0.6312**	0.6206	0.6183
2010	0.7100	0.5727	**0.7433**	0.5845	0.6234	0.5613	0.6228	0.6138	0.6098
2014	0.6676	0.5755	0.7455	0.5860	0.6257	0.5638	**0.6301**	0.6190	0.6178
					Canada (CA)				
1981	0.6159	0.5517	0.7265	0.5712	**0.6044**	0.5408	0.5744	0.5687	0.5653
1987	0.6571	0.5515	0.7265	0.5709	**0.6042**	0.5404	0.5846	0.5779	0.5744
1991	0.6450	0.5509	0.7261	0.5705	**0.6036**	0.5398	0.5872	0.5806	0.5770
1994	0.6285	0.5520	0.7270	0.5714	**0.6048**	0.5411	0.5861	0.5798	0.5758
1997	0.6368	0.5548	0.7293	0.5733	**0.6075**	0.5440	0.5851	0.5788	0.5751
1998	0.6418	0.5637	0.7366	0.5790	**0.6156**	0.5529	0.6016	0.5933	0.5911
2000	0.6497	0.5656	0.7383	0.5801	**0.6174**	0.5547	0.6150	0.6054	0.6034
2004	0.6370	0.5664	0.7388	0.5806	**0.6181**	0.5555	0.6139	0.6047	0.6021
2007	0.6356	0.5657	0.7384	0.5801	0.6174	0.5547	**0.6205**	0.6107	0.6082
2010	0.6184	0.5655	0.7382	0.5800	**0.6173**	0.5546	0.6160	0.6068	0.6043
2013	0.6248	0.5670	0.7389	0.5812	**0.6186**	0.5562	0.6033	0.5959	0.5915
					Germany (DE)				
1978	0.5630	0.5448	0.7215	**0.5657**	0.5973	0.5325	0.6226	0.6118	0.6100
1981	0.5985	0.5381	0.7146	0.5607	**0.5898**	0.5245	0.5958	0.5886	0.5852
1983	0.5310	0.5440	0.7206	0.5651	0.5964	**0.5315**	0.6159	0.6067	0.6031
1984	0.5959	0.5430	0.7192	0.5646	**0.5953**	0.5306	0.5857	0.5772	0.5768
1987	0.6004	0.5407	0.7171	0.5628	**0.5928**	0.5278	0.5913	0.5834	0.5813
1989	0.6089	0.5423	0.7188	0.5639	**0.5945**	0.5296	0.6029	0.5940	0.5926
1991	0.6348	0.5467	0.7227	0.5674	**0.5994**	0.5350	0.5962	0.5886	0.5846
1994	0.6303	0.5443	0.7205	0.5655	0.5967	0.5320	**0.5994**	0.5905	0.5880
1995	0.6264	0.5434	0.7198	0.5649	**0.5958**	0.5311	0.5951	0.5856	0.5844
1998	0.6262	0.5422	0.7186	0.5639	0.5945	0.5296	**0.5977**	0.5884	0.5860
2000	0.6196	0.5432	0.7197	0.5647	0.5956	0.5308	**0.6021**	0.5926	0.5912
2001	0.5540	**0.5486**	0.7249	0.5685	0.6012	0.5368	0.6223	0.6092	0.6102
2002	0.5551	**0.5473**	0.7236	0.5676	0.5999	0.5354	0.6111	0.6004	0.6001
2003	0.5550	**0.5483**	0.7245	0.5683	0.6009	0.5366	0.6121	0.6004	0.6016
2004	0.5680	0.5490	0.7252	**0.5688**	0.6016	0.5372	0.6194	0.6079	0.6075
2005	0.5829	0.5557	0.7310	**0.5734**	0.6083	0.5444	0.6317	0.6191	0.6192
2006	0.5837	0.5538	0.7293	**0.5721**	0.6064	0.5424	0.6258	0.6140	0.6132
2007	0.5906	0.5549	0.7304	0.5728	**0.6075**	0.5436	0.6301	0.6179	0.6181
2008	0.5936	0.5541	0.7296	0.5723	0.6067	0.5427	0.6264	0.6149	**0.6141**
2009	0.6481	0.5527	0.7282	0.5715	0.6054	0.5414	**0.6139**	0.6041	0.6025
2010	0.6472	0.5537	0.7290	0.5723	0.6064	0.5426	**0.6104**	0.6011	0.5991
2011	0.6517	0.5539	0.7293	0.5723	0.6066	0.5427	**0.6190**	0.6084	0.6066
2012	0.6542	0.5551	0.7302	0.5731	0.6077	0.5440	**0.6155**	0.6051	0.6035
2013	0.6456	0.5559	0.7309	0.5736	0.6084	0.5447	**0.6207**	0.6103	0.6086
2014	0.6459	0.5555	0.7304	0.5735	0.6081	0.5445	**0.6087**	0.5990	0.5969
2015	0.6340	0.5573	0.7321	0.5746	0.6098	0.5463	**0.6178**	0.6073	0.6064

Notes: The table report the empirical MPS (based on disposable income), together with the MPS implied by the eight parametric forms discussed in the article. The Lorenz curves are fitted to the 20 percentile data points and the implied MPS are calculated based on the mean-squared-error minimizing parameter(s) for each functional form. Highlighted in bold is the functional form which, out of all uni- and multiparametric forms, has an implied MPS that comes closest to the empirical MPS.

Table S-25: Empirical MPS and the MPS Implied by the Parametric Forms (Part 2)

	MPS	Logn	Pareto	Chot.	Rohde	Weib.	Wang/S.	Vi./Arn.	Kakwani
					Denmark (DK)				
1987	0.6292	0.5415	0.7176	0.5635	**0.5936**	0.5290	0.5660	0.5610	0.5607
1992	0.6290	0.5365	0.7125	0.5595	**0.5879**	0.5228	0.5573	0.5536	0.5529
1995	0.6309	0.5314	0.7072	0.5551	**0.5816**	0.5159	0.5678	0.5632	0.5616
2000	0.6258	0.5326	0.7085	0.5561	**0.5831**	0.5175	0.5684	0.5641	0.5622
2004	0.6253	0.5339	0.7099	0.5573	**0.5848**	0.5193	0.5676	0.5632	0.5613
2007	0.6315	0.5377	0.7141	0.5604	**0.5893**	0.5242	0.5783	0.5722	0.5722
2010	0.6460	0.5429	0.7192	0.5644	**0.5951**	0.5305	0.5866	0.5789	0.5796
2013	0.6546	0.5408	0.7173	0.5628	**0.5929**	0.5279	0.5942	0.5867	0.5855
					Spain (ES)				
1980	0.6315	0.5669	0.7392	0.5809	0.6185	0.5559	**0.6213**	0.6115	0.6077
1985	0.6227	0.5650	0.7381	0.5795	0.6169	0.5539	0.6339	**0.6225**	0.6193
1990	0.6338	0.5602	0.7341	0.5766	0.6126	0.5493	**0.6172**	0.6083	0.6038
1995	0.6376	0.5833	0.7504	0.5907	0.6320	0.5708	**0.6300**	0.6199	0.6154
2000	0.6503	0.5743	0.7444	0.5855	**0.6248**	0.5628	0.6238	0.6148	0.6102
2004	0.6375	0.5648	0.7372	0.5799	**0.6167**	0.5541	0.5991	0.5924	0.5875
2007	0.6319	0.5619	0.7348	0.5780	**0.6140**	0.5513	0.5885	0.5816	0.5780
2010	0.6166	0.5728	0.7427	0.5850	**0.6235**	0.5618	0.5909	0.5837	0.5796
2013	0.5944	0.5770	0.7458	0.5874	0.6269	0.5655	0.6033	**0.5957**	0.5911
					Finland (FI)				
1987	0.4876	0.5269	0.7018	0.5510	0.5756	**0.5095**	0.5559	0.5520	0.5502
1991	0.5266	**0.5275**	0.7026	0.5516	0.5765	0.5104	0.5577	0.5529	0.5514
1995	0.5386	**0.5299**	0.7059	0.5536	0.5797	0.5135	0.5906	0.5830	0.5815
2000	0.5033	0.5418	0.7187	0.5634	0.5940	**0.5289**	0.6166	0.6072	0.6065
2004	0.5703	0.5453	0.7220	**0.5660**	0.5977	0.5330	0.6200	0.6100	0.6094
2007	0.5483	0.5454	0.7219	0.5663	0.5979	**0.5333**	0.6081	0.5994	0.5983
2010	0.5620	0.5444	0.7208	**0.5655**	0.5968	0.5322	0.6041	0.5963	0.5941
2013	0.5429	**0.5437**	0.7202	0.5649	0.5960	0.5312	0.6065	0.5982	0.5959
					Israel (IS)				
1979	0.5253	0.5604	0.7340	0.5769	0.6128	**0.5497**	0.6075	0.6019	0.5948
1986	0.5629	0.5631	0.7361	0.5786	0.6152	**0.5523**	0.6124	0.6059	0.5987
1992	0.5825	0.5612	0.7347	**0.5774**	0.6135	0.5504	0.6124	0.6058	0.5988
1997	0.5883	0.5747	0.7445	**0.5858**	0.6251	0.5633	0.6199	0.6124	0.6061
2001	0.5823	**0.5817**	0.7493	0.5899	0.6307	0.5695	0.6250	0.6171	0.6112
2005	0.5832	0.5941	0.7569	0.5969	0.6400	**0.5799**	0.6272	0.6192	0.6131
2007	0.5851	**0.5900**	0.7543	0.5948	0.6370	0.5767	0.6188	0.6122	0.6051
2010	0.5864	0.5980	0.7593	0.5989	0.6428	**0.5830**	0.6323	0.6244	0.6179
2012	0.5900	**0.5915**	0.7552	0.5956	0.6382	0.5779	0.6186	0.6112	0.6048
2014	0.6027	0.5874	0.7524	0.5935	0.6350	0.5746	**0.6030**	0.5961	0.5910
2016	0.5854	0.5787	0.7467	0.5885	0.6283	0.5671	0.5992	0.5934	**0.5872**

Notes: The table is the continuation of Table S-24.

Table S-26: Empirical MPS and the MPS Implied by the Parametric Forms (Part 3)

	MPS	Logn	Pareto	Chot.	Rohde	Weib.	Wang/S.	Vi./Arn.	Kakwani
					Italy (IT)				
1986	0.5944	0.5624	0.7358	0.5780	0.6145	0.5515	0.6182	0.6101	**0.6052**
1987	0.5533	0.5728	0.7433	0.5846	0.6236	**0.5615**	0.6238	0.6151	0.6089
1989	0.6036	0.5606	0.7344	0.5769	0.6129	0.5497	0.6178	**0.6098**	0.6043
1991	0.6172	0.5549	0.7296	0.5732	**0.6075**	0.5439	0.6010	0.5945	0.5896
1993	0.6155	0.5757	0.7452	0.5864	0.6259	0.5641	**0.6157**	0.6074	0.6030
1995	0.6125	0.5749	0.7448	0.5859	0.6252	0.5634	**0.6170**	0.6081	0.6052
1998	0.6039	0.5767	0.7462	0.5868	0.6266	0.5649	0.6242	0.6137	**0.6123**
2000	0.6024	0.5705	0.7419	0.5831	0.6216	0.5593	0.6216	0.6122	**0.6093**
2004	0.6206	0.5731	0.7439	0.5845	0.6237	0.5615	0.6343	0.6233	**0.6215**
2008	0.6162	0.5676	0.7399	0.5812	0.6191	0.5565	0.6256	0.6162	**0.6131**
2010	0.5946	0.5662	0.7385	0.5805	0.6179	0.5553	0.6110	0.6023	**0.5993**
2014	0.5778	0.5663	0.7384	**0.5807**	0.6179	0.5554	0.6032	0.5958	0.5923
					Luxembourg (LU)				
1985	0.5348	**0.5358**	0.7121	0.5588	0.5872	0.5217	0.5921	0.5857	0.5805
1991	0.5933	0.5367	0.7132	0.5595	0.5882	0.5228	0.5975	**0.5910**	0.5867
1994	0.6045	0.5358	0.7122	0.5588	0.5871	0.5215	0.5974	**0.5909**	0.5859
1997	0.5843	0.5441	0.7205	0.5653	0.5966	0.5318	0.6089	0.6007	**0.5964**
2000	0.5905	0.5447	0.7211	0.5658	0.5973	0.5325	0.6106	0.6032	**0.5975**
2004	0.6129	0.5471	0.7233	0.5675	0.5997	0.5352	**0.6124**	0.6024	0.5998
2007	0.6383	0.5498	0.7259	0.5693	0.6024	0.5381	**0.6235**	0.6129	0.6111
2010	0.5917	0.5479	0.7238	0.5682	0.6006	0.5362	0.6029	**0.5957**	0.5911
2013	0.5670	0.5529	0.7280	**0.5718**	0.6056	0.5418	0.5988	0.5904	0.5876
					Mexico (MX)				
1984	0.6347	**0.6287**	0.7766	0.6140	0.6633	0.6055	0.6765	0.6644	0.6573
1989	0.6672	**0.6590**	0.7918	0.6273	0.6812	0.6252	0.7154	0.6986	0.6971
1992	0.7334	0.6784	0.8004	0.6359	0.6919	0.6373	**0.7267**	0.7103	0.7075
1994	0.7401	0.6822	0.8019	0.6377	0.6939	0.6397	**0.7251**	0.7095	0.7055
1996	0.7139	0.6711	0.7971	0.6328	0.6879	0.6329	**0.7199**	0.7042	0.7005
1998	0.7042	0.6798	0.8006	0.6370	0.6925	0.6383	0.7121	**0.6971**	0.6924
2000	0.7208	0.6762	0.7991	0.6354	0.6906	0.6361	**0.7110**	0.6964	0.6913
2002	0.7087	0.6582	0.7911	0.6273	0.6808	0.6249	**0.7031**	0.6886	0.6832
2004	0.6479	**0.6550**	0.7897	0.6258	0.6789	0.6229	0.7035	0.6885	0.6841
2008	0.6640	**0.6692**	0.7960	0.6324	0.6868	0.6318	0.7006	0.6848	0.6820
2010	0.6791	0.6510	0.7876	0.6244	**0.6766**	0.6204	0.6859	0.6705	0.6678
2012	0.7026	0.6564	0.7902	0.6267	0.6797	0.6238	**0.6942**	0.6783	0.6757
					Netherlands (NL)				
1983	0.6127	0.5404	0.7168	0.5626	**0.5925**	0.5275	0.5900	0.5815	0.5797
1987	0.6070	0.5356	0.7119	0.5587	0.5869	0.5214	**0.5884**	0.5813	0.5778
1990	0.5984	0.5468	0.7229	0.5673	**0.5994**	0.5350	0.6001	0.5902	0.5899
1993	0.5556	0.5439	0.7193	0.5656	0.5963	0.5321	**0.5507**	0.5441	0.5429
1999	0.5726	0.5339	0.7101	0.5573	0.5848	0.5192	0.5804	**0.5740**	0.5710
2004	0.5615	0.5472	0.7235	**0.5676**	0.5998	0.5353	0.6075	0.5956	0.5973
2007	0.5688	**0.5501**	0.7265	0.5694	0.6027	0.5383	0.6377	0.6240	0.6253
2010	0.5305	0.5440	0.7205	0.5652	0.5964	**0.5317**	0.6044	0.5942	0.5931
2013	0.5291	**0.5455**	0.7219	0.5663	0.5980	0.5333	0.6094	0.5988	0.5983

Notes: The table is the continuation of Table S-24.

Table S-27: Empirical MPS and the MPS Implied by the Parametric Forms (Part 4)

	MPS	Logn	Pareto	Chot.	Rohde	Weib.	Wang/S.	Vi./Arn.	Kakwani
					Norway (NO)				
1979	0.4789	0.5330	0.7088	0.5565	0.5836	**0.5180**	0.5638	0.5574	0.5571
1986	0.5532	0.5346	0.7108	**0.5579**	0.5857	0.5202	0.5748	0.5684	0.5667
1991	0.4935	0.5337	0.7100	0.5570	0.5845	**0.5189**	0.5829	0.5757	0.5751
1995	0.5505	0.5368	0.7133	**0.5596**	0.5882	0.5229	0.5856	0.5782	0.5787
2000	0.5423	**0.5429**	0.7200	0.5640	0.5950	0.5300	0.6269	0.6125	0.6192
2004	0.6152	0.5506	0.7275	0.5694	**0.6031**	0.5385	0.6585	0.6364	0.6513
2007	0.6403	0.5378	0.7143	0.5604	**0.5894**	0.5242	0.5855	0.5769	0.5780
2010	0.6462	0.5393	0.7159	0.5616	**0.5911**	0.5261	0.5866	0.5773	0.5794
2013	0.6405	0.5398	0.7162	0.5620	0.5917	0.5267	**0.5849**	0.5762	0.5770
					Poland (PL)				
1986	0.6151	0.5471	0.7229	0.5677	**0.5998**	0.5355	0.5885	0.5827	0.5787
1992	0.6417	0.5445	0.7210	0.5655	0.5970	0.5321	**0.6166**	0.6072	0.6037
1995	0.5853	0.5670	0.7393	**0.5809**	0.6185	0.5559	0.6111	0.5983	0.6008
1999	0.6045	0.5565	0.7314	0.5740	**0.6090**	0.5454	0.6161	0.6036	0.6050
2004	0.6004	0.5694	0.7413	0.5823	0.6206	0.5580	0.6288	**0.6151**	0.6165
2007	0.6387	0.5664	0.7393	0.5803	0.6180	0.5551	**0.6381**	0.6240	0.6249
2010	0.6365	0.5658	0.7387	0.5800	0.6175	0.5546	**0.6320**	0.6190	0.6187
2013	0.6316	0.5704	0.7420	0.5829	0.6214	0.5590	0.6273	**0.6134**	0.6153
2016	0.6426	0.5567	0.7315	0.5742	0.6092	0.5457	**0.6102**	0.5974	0.5991
					Taiwan (TW)				
1981	0.6056	0.5466	0.7231	0.5671	0.5993	0.5346	0.6252	0.6156	**0.6105**
1986	0.6232	0.5478	0.7243	0.5679	0.6005	0.5358	0.6324	0.6218	**0.6176**
1991	0.6224	0.5484	0.7246	0.5684	0.6011	0.5366	**0.6209**	0.6120	0.6067
1995	0.6190	0.5523	0.7278	0.5713	0.6050	0.5410	**0.6144**	0.6046	0.6012
1997	0.6110	0.5539	0.7291	0.5723	**0.6066**	0.5427	0.6197	0.6106	0.6059
2000	0.6155	0.5545	0.7297	0.5727	0.6072	0.5433	0.6206	**0.6109**	0.6067
2005	0.6453	0.5610	0.7350	0.5771	0.6133	0.5500	**0.6279**	0.6176	0.6132
2007	0.6397	0.5618	0.7354	0.5776	0.6140	0.5508	**0.6246**	0.6148	0.6097
2010	0.6364	0.5656	0.7382	0.5801	0.6174	0.5546	**0.6209**	0.6110	0.6071
2013	0.6443	0.5619	0.7355	0.5776	0.6141	**0.5509**	0.6225	0.6117	0.6085
2016	0.6519	0.5601	0.7342	0.5765	0.6125	**0.5491**	0.6228	0.6128	0.6087
					United Kingdom (UK)				
1979	0.6088	0.5459	0.7216	0.5669	**0.5985**	0.5342	0.5826	0.5773	0.5727
1986	0.6110	0.5611	0.7344	0.5775	**0.6134**	0.5505	0.5973	0.5899	0.5858
1991	0.6344	0.5762	0.7457	0.5867	**0.6264**	0.5646	0.6227	0.6147	0.6093
1994	0.6764	0.5794	0.7483	0.5882	0.6289	0.5672	**0.6438**	0.6333	0.6289
1995	0.6322	0.5796	0.7480	0.5885	**0.6290**	0.5675	0.6281	0.6189	0.6144
1999	0.6656	0.5850	0.7517	0.5915	0.6332	0.5721	**0.6384**	0.6274	0.6242
2004	0.6945	0.5841	0.7517	0.5907	0.6325	0.5710	**0.6588**	0.6452	0.6438
2007	0.6795	0.5779	0.7473	0.5873	0.6276	**0.5658**	0.6427	0.6307	0.6285
2010	0.6845	0.5756	0.7459	0.5858	0.6258	0.5636	**0.6514**	0.6381	0.6365
2013	0.6813	0.5738	0.7446	0.5848	0.6243	0.5620	**0.6476**	0.6356	0.6326
					United States (US)				
1979	0.5924	0.5619	0.7349	0.5781	0.6141	0.5514	**0.5861**	0.5799	0.5764
1986	0.5927	0.5752	0.7445	0.5864	0.6255	0.5640	**0.5960**	0.5898	0.5851
1991	0.6020	0.5778	0.7464	0.5878	0.6276	0.5662	**0.6052**	0.5982	0.5936
1994	0.6225	0.5901	0.7549	0.5944	0.6370	0.5764	0.6348	0.6243	**0.6216**
1997	0.6340	0.5912	0.7558	0.5949	**0.6379**	0.5772	0.6425	0.6308	0.6293
2000	0.6370	0.5898	0.7550	0.5940	**0.6368**	0.5760	0.6437	0.6314	0.6309
2004	0.6343	0.5915	0.7558	0.5951	0.6380	0.5775	**0.6374**	0.6262	0.6246
2007	0.6306	0.5959	0.7584	0.5976	0.6413	0.5811	0.6371	**0.6254**	0.6241
2010	0.6225	0.5898	0.7545	0.5944	0.6368	0.5763	0.6280	**0.6187**	0.6144
2013	0.6351	0.5952	0.7580	0.5972	0.6408	0.5806	**0.6394**	0.6288	0.6249
2016	0.6521	0.5971	0.7592	0.5982	0.6422	0.5821	**0.6443**	0.6326	0.6293

Notes: The table is the continuation of Table S-24.
